# Surfactant encapsulated palladium-polyoxometalates: controlled assembly and their application as single-atom catalysts†
†Electronic supplementary information (ESI) available. See DOI: 10.1039/c5sc03554f


**DOI:** 10.1039/c5sc03554f

**Published:** 2015-10-26

**Authors:** Peilei He, Biao Xu, Xiaobin Xu, Li Song, Xun Wang

**Affiliations:** a Department of Chemistry , Tsinghua University , Beijing , 100084 , China . Email: wangxun@mail.tsinghua.edu.cn; b National Synchrotron Radiation Laboratory , University of Science and Technology of China , Hefei , 230029 , China

## Abstract

The challenge with single-atom catalysts (SACs) is in designing a highly definite structure with accurate location of the single atom and high catalytic efficiency. The noble metal substituted polyoxometalates seem to be a kind of SAC because of their well resolved crystal structure. Here, we got two kinds of assembly structures (nanorolls and hollow spindles) based on the palladium substituted Wells–Dawson polyoxometalate (Pd-POM), which consists of isolated Pd atoms. Both the nanorolls and hollow spindles showed high catalytic activity for both the Suzuki–Miyaura coupling reaction and semihydrogenation reaction. The difference of the chemical surroundings between the nanorolls and hollow spindles leads to their discrepancy in the catalytic activity of semihydrogenation.

## Abstract 2

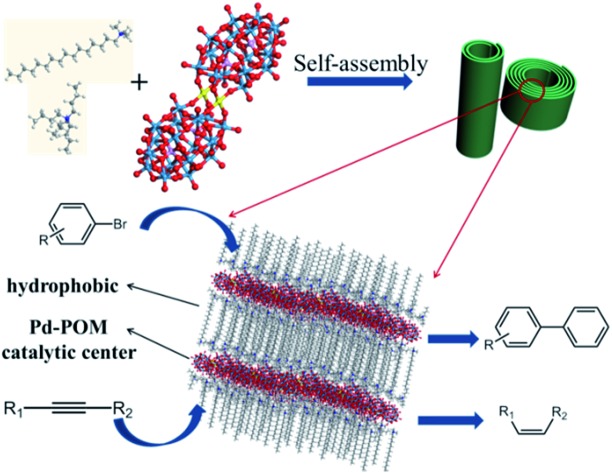
Two kinds of assembly structures (nanorolls and hollow spindles) based on the palladium substituted Wells–Dawson polyoxometalate (Pd-POM) were synthesised and showed high catalytic activity for both the Suzuki–Miyaura coupling reaction and semihydrogenation reaction.

## Introduction

Supported metal catalysts as a kind of heterogeneous catalyst have been widely used in industry because of their higher catalytic efficiencies. However, in traditional supported noble metal catalysts, only a small portion of noble metal atoms act as the active center.^1^ To improve the atom efficiency, single-atom catalysts (SACs) are the most promising catalysts which can decrease the use of noble metals and increase the catalytic efficiency to save energy.^2^–^4^ In recent advances, most of the single atoms were anchored to metal oxides,^5^,^6^ metal surfaces,^7^ and graphene^8^ through a mass-selected soft-landing technique^9^ or wet chemistry method.^10^ The single noble metal atoms on the supports can only be confirmed using subangstrom-resolution scanning transmission electron microscopy (STEM).^11^,^12^ Even so, the location and coordination environment of the single atom is unpredictable in the catalyst.

How to get a SAC with an accurate location and coordination environment for the noble metal is still a major challenge. Based on our understanding of polyoxometalates (POMs), we found that one noble metal atom can be incorporated into POMs as a heteroatom.^13^ And the crystal structures of the noble metal substituted POMs are usually well resolved. There is a series of noble metal substituted POMs in the literature.^13^ Therefore, this kind of POM can be a good candidate for SACs with an explicit component, structure and coordination environment. What's more, the structural diversity of POMs provides a chance to discover the new large class of SAC.

However, the direct use of pure polyoxometalates is often associated with difficult catalyst recovery^14^,^15^ and lower efficiency in organic catalytic reactions. A much simpler method is to encapsulate the POMs with surfactants.^16^,^17^ The surfactant encapsulated POMs (SEPs) are much easier to recover and show better dispersibility in organic solvents.^18^,^19^ Meanwhile, the chemical surrounding of POMs can be easily tuned based on the choice of surfactants. In particular, the surface properties of the SEPs will be changed. So, through this method, numerous heterogeneous single atom catalysts could be obtained based on all kinds of POMs.

**Fig. 1:**
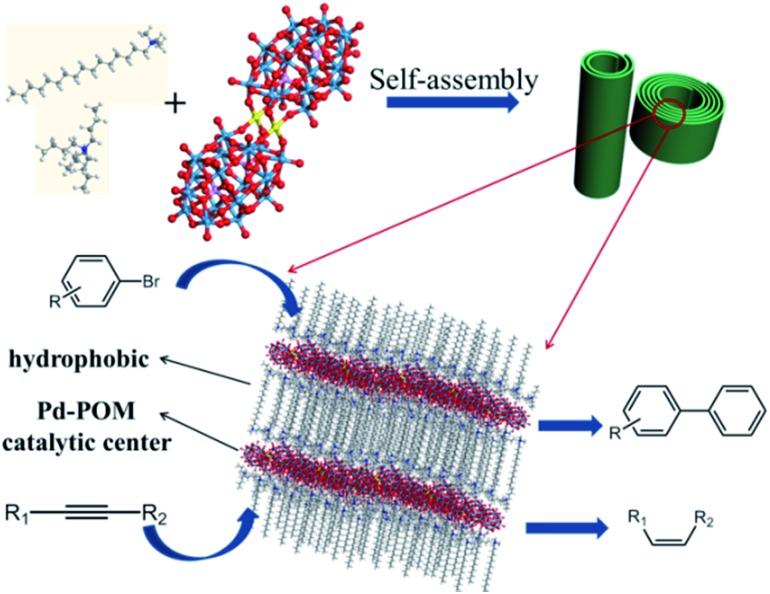
Schematic illustration of the Pd-POM nanorolls and the two kinds of catalytic reactions.

Palladium compounds are often used as catalysts in many organic reactions, such as coupling reactions,^20^ hydrogenation,^21^ and selective oxidation.^22^ Therefore, in this work, we present a nice study on the controlled assembly of a palladium substituted Wells–Dawson POM^23^ (Pd-POM) with different surfactants and its application as a single-atom catalyst. And this surfactant encapsulated Pd-POM could assemble into nanorolls and hollow spindles. When the Pd-POM was encapsulated by cetrimonium bromide (CTAB) and tetrabutylammonium bromide (TBAB), we observed some nanorolls with different length–diameter (*L*/*D*) ratios and coiling cycles (Fig. 1). Some hollow spindles were obtained when the surfactants were decyltrimethylammonium bromide (DTAB) and tetraethylammonium bromide (TEAB). Most importantly, the as obtained assemblies could act as high-performance catalysts in both Suzuki–Miyaura coupling reactions and semi-hydrogenation of alkynes with a TOF as high as above 2000 h^–1^ and stereo-selectivity of *Z*/*E* > 99/1. The current concept and results are very important and would be very enlightening in both the materials science and catalysis fields.

## Results and discussion

**Fig. 2:**
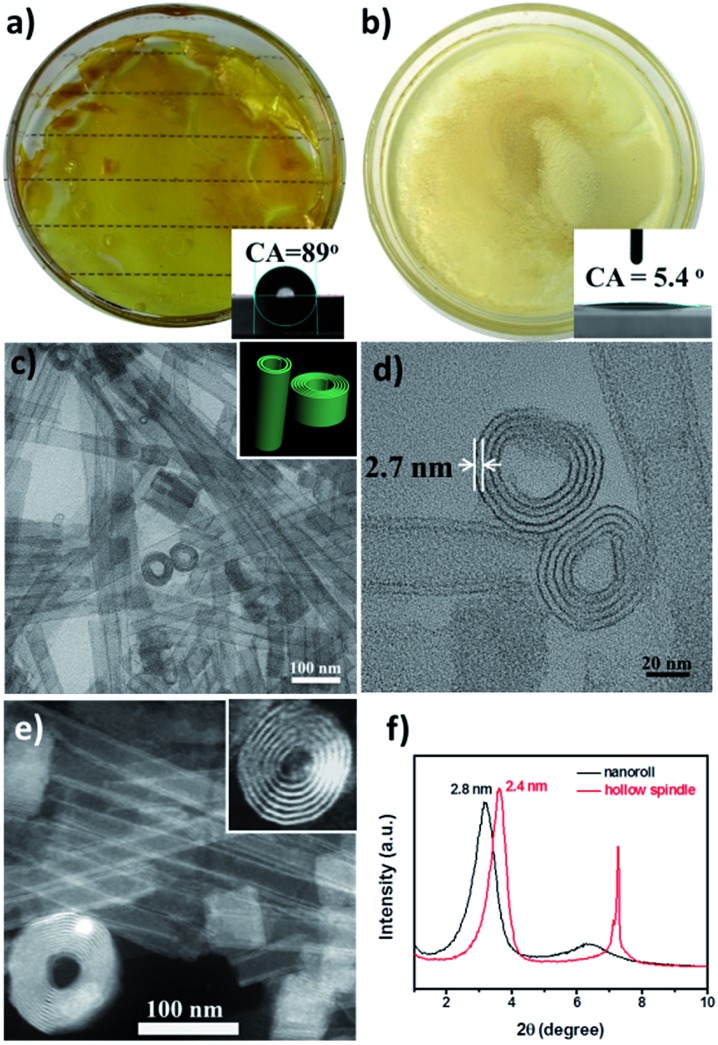
(a) The photo image of the film which was composed of the nanorolls. Inset: the static contact angle of the film. (b) The photo image of the spindle powders. Inset: the static contact of the spindle. (c) TEM image of the nanorolls. Inset: the scheme of the nanorolls with different *L*/*D* ratios. (d) HRTEM image of the nanorolls. (e) STEM image of the nanorolls. Inset: a magnified image of a nanoroll. (f) SAXRD of the nanorolls and hollow spindles.

First, we have synthesized the Pd-POM (K_15_[Pd_2_(α_2_-P_2_W_17_O_61_)_2_H]) according to ref. 23. The structure of this kind of Pd-POM can be confirmed using ^31^P NMR, mass spectrometry and Fourier transform infrared (FTIR) characterization (Fig. S1 and S2†). The chemical composition of the Pd-POM was determined using inductively coupled plasma optical emission spectrometry (ICP-OES). And the experimental data (K, 6.359; P, 1.118; Pd, 2.631; W, 64.34) was very close to the calculated data (K, 6.426; P, 1.357; Pd, 2.332; W, 68.49). X-ray photoelectron spectroscopy (XPS) revealed that the valence state of Pd is +2 (Fig. S3†). Then a two-phase method^16^ was used to get the SEPs. And we still employed two kinds of surfactants.^24^,^25^ When the surfactants were CTAB and TBAB, a yellow film (as Fig. 2a shows) was observed after the evaporation of chloroform. Then the film was dissolved in chloroform and some nanoroll structures formed (Fig. S4†). After adjusting the volume ratio of chloroform and acetone to 2 : 1, pure nanoroll structures were obtained as shown in Fig. 2c and d. Then the pure nanoroll sample could be obtained when the organic solvent was completely evaporated. From the TEM image (Fig. 2c), there were some nanorolls with different *L*/*D* ratios. The STEM result (Fig. 2e) showed the structure of the nanorolls much more clearly. And some bundles of nanorolls could be found. When some individual nanorolls were standing, a cross-section could be easily observed. And we have investigated the nanorolls using small-angle X-ray diffraction (SAXRD) characterization. The interlayer spacing was about 2.8 nm (Fig. 2f) which corresponded to the result of the HRTEM image (Fig. 2d). The formation mechanism of the nanorolls should be the same as the mechanism that we discussed in a previous paper.^24^ And the composition of the nanorolls was fully characterized using FTIR (Fig. S5a†), thermogravimetric analysis (TGA, Fig. S5b†) and C, H, and N elemental analysis (Fig. S6†). The TGA data indicated the full substitution of organic cations. The contact angle of the nanorolls was about 89° (the inset in Fig. 2a), which shows a little hydrophobicity. When we used DTAB and TEAB, we got a hollow spindle structure (Fig. S7†) in the aqueous phase. The hollow spindle sample (Fig. 2b) was obtained after the water was completely evaporated. The length of the hollow spindles was about 500 nm (Fig. S7a†). Lamellar structures were observed in the magnified TEM image (Fig. S7b†) and were confirmed using SAXRD characterization (Fig. 2f). The hollow structure was very obvious in the STEM image (Fig. S7c†). The contact angle of the spindle-like structure was about 5.4° (the inset in Fig. 2b), caused by the decrease in the chain length of the surfactants. The difference between the contact angles of the nanorolls and hollow spindles leads to the difference in the formation of a film (Fig. 2a) or precipitate (Fig. 2b). The FTIR spectrum (Fig. S8a†) proved the existence of the surfactants. Also, the TGA (Fig. S8b†) and C, H, and N elemental analysis (Fig. S6†) were done. The energy-dispersive X-ray (EDX) mapping (Fig. S9†) analysis of these two kinds of SACs confirmed the even distribution of nitrogen, phosphorous, tungsten and palladium.

**Fig. 3:**
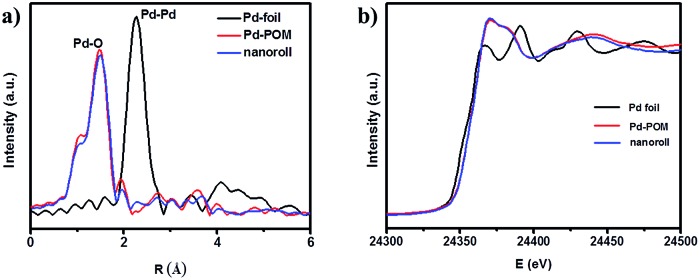
(a) Fourier-transformed spectra of *k*^3^-weighted Pd K-edge EXAFS of the Pd foil, Pd-POM and nanorolls. (b) The normalized XANES spectra at the Pd K-edge of the Pd foil, Pd-POM and nanoroll.

To verify that the Pd-POM contained only atomically dispersed single Pd atoms as the crystal structure shows (Fig. 1), extended X-ray absorption fine structure (EXAFS) spectra were measured on the pure Pd-POM and nanoroll structures. Indeed, in the Fourier-transformed EXAFS spectra (*r* space, Fig. 3a), there was only one peak at ∼1.5 Å from the Pd–O contribution in the Pd-POM and no peak at ∼2.26 Å from the Pd–Pd contribution. The EXAFS data did not reveal any Pd–Pd contribution in the Pd-POM, in agreement with the fact that there were only Pd–O bonds in the crystal structure. And the X-ray absorption near-edge structure (XANES) spectra of the Pd-POM and nanorolls, and the reference spectra of the Pd foil are shown in Fig. 3b. The whiteline intensities of Pd in the Pd-POM and nanorolls were almost exactly the same. Both of them were higher than the intensity of the Pd foil. The whiteline intensity reflects the oxidation state of Pd in the different samples.^26^ So this result suggested that the Pd single atoms in the Pd-POM carry positive charges. These results further proved that the nanorolls and hollow spindles are SACs.

According to ref. 27, the Pd^2+^ complexes are active in the Suzuki–Miyaura coupling reaction. Therefore, to test the catalytic activity of the SACs that we obtained, the Suzuki–Miyaura coupling reaction was selected as the model reaction. The Suzuki–Miyaura coupling reaction involves the coupling of an aryl halide (including iodide, bromide and chloride) and an aromatic boronic acid. In general, aryl iodides are the most active substrates, and aryl chlorides are the most inert ones. Meanwhile, the room-temperature (RT) reaction between an aryl bromide and phenylboronic acid has been reported.^28^,^29^ For this reason, we chose an aryl bromide and RT to perform the Suzuki–Miyaura coupling reaction. After optimization, we selected C_2_H_5_OH–H_2_O (1 : 1 mL) as the co-solvent, TBAB as the phase-transfer catalyst and K_2_CO_3_ as a base for the catalytic reaction. The amount of catalyst was tested to determine the optimum reaction conditions. And we found that 0.13 mol% Pd (6.4 × 10^–7^ mol, combined with the ICP result, Fig. S10†) was sufficient to obtain an excellent yield of 4-acetylbiphenyl (96%, Table S1,† entry 1) within 20 min, at 303 K. The cross-coupling reaction was completed within 20 min, resulting in a turnover frequency (TOF) up to 2250 h^–1^.

In order to see the catalytic efficiency of this catalytic system, a series of aryl halides were used as substrates to carry out the coupling reaction under the optimized conditions (Table S1†). High yields from 80% to 97% were obtained for the different substrates. All of the products were purified using column chromatography and verified using ^1^H NMR and ^13^C NMR (in the ESI†). Compared with 4-bromoanisole (Table S1,† entry 5) and 4-bromoacetophenone (Table S1,† entry 1), we could see that the stronger the electron-donating group in the aryl bromide, the lower the catalytic activity. Although the steric hindrance of 3-bromobenzaldehyde was increased, the decrease in yield was not obvious (Table S1,† entry 13). The hollow spindles were also used to catalyze the Suzuki–Miyaura reaction. There might be a difference of chemical surroundings between the nanoroll and the hollow spindle structures according to their different contact angles. However, the hollow spindle structure showed similar catalytic activity to the nanoroll structure (Table S1†). The main reason was that in the ethanol–water system we used both the nanoroll and hollow spindle structures which have similar dispersibility and thus achieve similar catalytic efficiency. Control experiments with (CTA)_3_(TBA)_3_P_2_W_18_O_62_ and (CTA)_*x*_(TBA)_(10–*x*)_P_2_W_17_O_61_ were also carried out under the same conditions. As the reaction time was prolonged to 12 hours, there was still no coupling product. Therefore, the catalytic active center is the Pd atom in the SEPs.

**Fig. 4:**
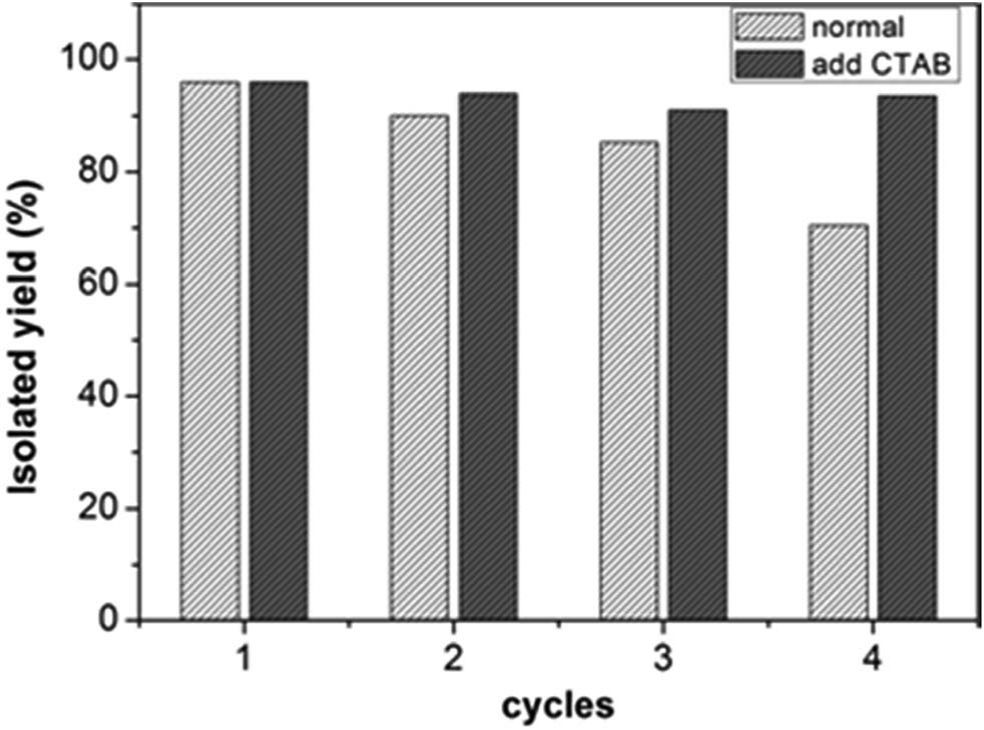
Catalytic experiment of recycled nanorolls for the Suzuki–Miyaura coupling reaction without or with addition of CTAB.

A great advantage of the supported metal catalyst is the repeatable use of catalysts. Therefore, a recycling experiment was performed with the catalysts being the nanorolls. The structure of the Pd-POM could be maintained according to FTIR and ^31^P NMR characterization (Fig. S11b and S11c†), though the morphology of the nanorolls changed after the catalytic reaction (Fig. S11a†). And the catalyst could be recycled several times (Fig. 4). There was an apparent loss of its high catalytic performance at the fourth cycle. Comparing the FTIR spectrum before and after the catalytic reaction, the apparent change was the decrease in the band at 2925 cm^–1^. The band was corresponding to methylene which was from CTAB and TBAB. So we could speculate that the surfactants in the SEP stripped from the surface of the POM during the reaction. Considering that TBAB was added into the reaction system, we have tried to add CTAB into the solution before the catalyst was reused. And we measured the loss of catalyst by weighing the mass of the catalyst. As Fig. S12† shows, the results indicate that the loss of catalyst is more obvious without the addition of CTAB. Amazingly, the high catalytic performance can be kept after four cycles (Fig. 4). In the control experiment, we determined that the yield of 4-bromoacetophenone with the bare Pd-POM is 36% (entry 3, Table S1†). It was much lower than with the nanoroll or the hollow spindle structures. These results indicated that the surfactants have positive effects on the Suzuki–Miyaura reaction. A more likely reason is that the aryl bromide and phenylboronic acid are organic. The hydrophobic alkyl chains of the surfactants on the surface of the nanorolls and hollow spindles contributed to enhance the probability of the interaction between the organic reactants and the catalytic center (Pd-POM). Also, the surfactants can decrease the loss of Pd-POM in the aqueous solution. For the bare Pd-POM, there is no surfactant on the surface which is adverse to the contact between the reactants and the bare Pd-POM. Some previous reports about SEP-based reusable catalysts support our results.^30^,^31^

Encouraged by the high activity of these SACs, we tried to examine their catalytic efficiency in other reactions. Pd compounds have also been studied for the semihydrogenation of alkynes with H_2_ but suffer from lower *Z*-selectivity and over-reduction of alkenes.^32^,^33^ According to ref. 34, the highly selective semihydrogenation of alkynes using organosilanes with water as a hydrogen source has been reported. Compared with hydrogen gas, organosilanes are a more promising hydrogen source because they are more prone to reaction under mild conditions. So we used the nanorolls and hollow spindles to catalyze semihydrogenation by using organosilanes as the hydrogen source. First, according to ref. 34, we know that the solvent has a great effect on the catalytic activity. After screening the solvents, we found that chloroform was the only suitable solvent and could afford a high yield in the reaction. It was sufficient to use 0.65 mol% Pd to obtain an excellent yield of *cis*-stilbene (87%, *Z*/*E* > 99/1, Table 1, entry 1) within 30 min, at 298 K. The nanorolls were further used to catalyze various internal alkynes under the same conditions. For all of these reactions, higher yields from 86% to 91% and a higher stereoselectivity (*Z*/*E* > 99/1) were obtained. In this case, the Pd-POM could be seen as a kind of shape selective catalyst. As Fig. S13† shows, the reactant approached the Pd atoms in the semi-open space of the center of the Pd-POM. The two Pd atoms were located on the same side of the reactant. Therefore, the hydrogenation reaction was more inclined to happen on one side of the reactant. Finally, most of the semihydrogenation products were *cis*-form products. After the catalytic reaction, some nanodisks appeared instead of the nanorolls (Fig. S14a†). The structure of the Pd-POM remained unchanged according to FTIR spectra (Fig. S14b†) in spite of the change of morphology. In addition, the catalyst could be recycled through centrifugation and used for another cycle without an obvious loss of catalytic activity (Table 1, entry 5, in parentheses). Then the catalytic activity of the hollow spindle structure was also examined with different internal alkynes. In Table 1, we can see that the nanorolls show much higher catalytic activity than the hollow spindles. In the control experiment (Table 1, entry 7), we showed that the catalytic activity of the bare Pd-POM was similar to that of the hollow spindles. These apparent differences should be caused by the difference of chemical surroundings that we have mentioned before. When the Pd-POM is encapsulated by CTAB and TBAB, the nanorolls show a comparatively large contact angle (89°) which leads them to disperse better in the organic solvents than the hydrophilic hollow spindles or the bare Pd-POM.

**Table 1:** The scope of the semihydrogenation reactions^*a*^

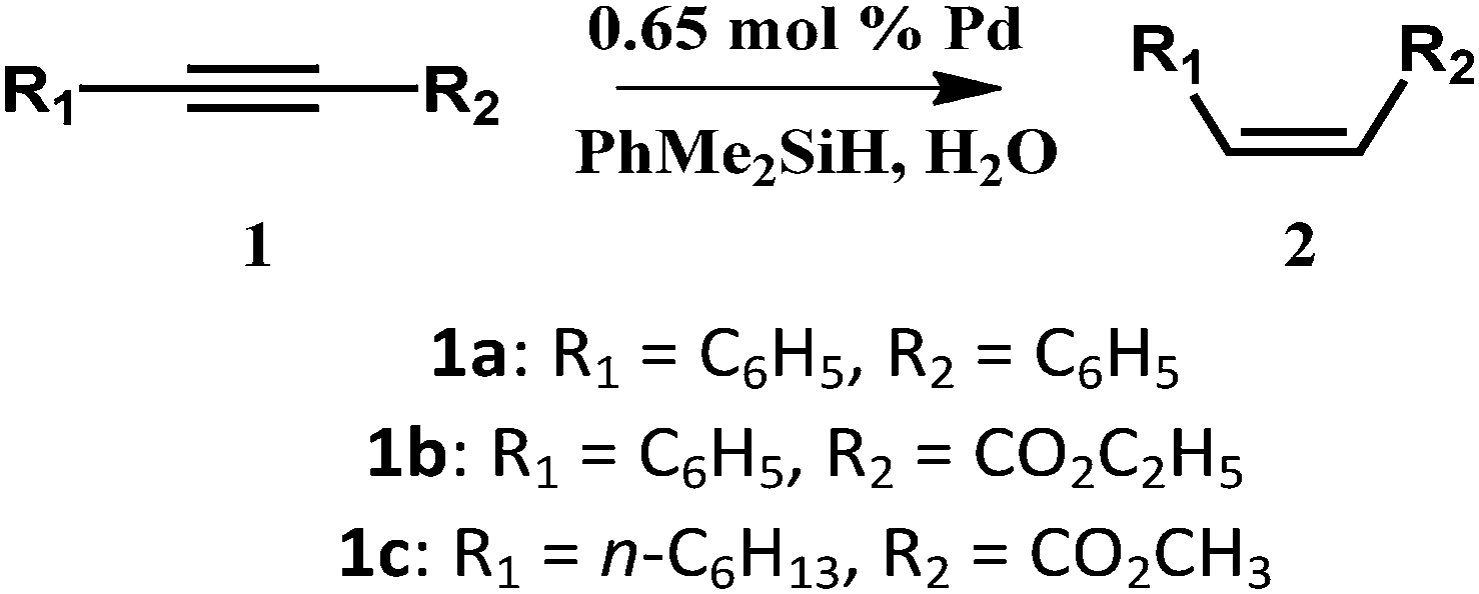				
Entry	Catalyst	**1**	Yield^*b*^ [%]	*Z*/*E*^*c*^
1	Nanoroll	**1a**	87	>99/1
2	Spindle	**1a**	37	>99/1
3	Nanoroll	**1b**	87	>99/1
4	Spindle	**1b**	12	>99/1
5^*d*^	Nanoroll	**1c**	91 (90^*e*^)	>99/1
6	Spindle	**1c**	67	>99/1
7	Pd-POM	**1c**	64	>99/1

^*a*^Unless otherwise noted, the reactions were carried out with **1** (0.1 mmol), dimethylphenylsilane (0.2 mmol), and water (10 μL) in the presence of the catalyst in CHCl_3_ (2 mL) at 25 °C for 30 min.

^*b*^ ^1^H NMR yield, using CH_2_Br_2_ as an internal standard.

^*c*^ *Z*/*E* ratio was determined using ^1^H NMR analysis.

^*d*^The reaction time was 15 min.

^*e*^The yield of the second use. All of the yields and *Z*/*E* ratios are in accordance with the GC-MS results.

Besides, we carried out leaching experiments to clarify whether palladium leached into the reaction system (Fig. S15†). After 10 min (the yield is 23%), 1 mL of the reaction solution was filtered and the supernatant was transferred to another vessel. The supernatant and the residual reaction solution were continuously stirred for 20 min. Though the yield of the residue was 81%, the yield of the supernatant was still 23%. These results indicate that the semihydrogenation reaction is catalyzed by the solid catalyst.

## Conclusions

In summary, we have found a new kind of SAC which was based on palladium substituted polyoxometalates. Through the surfactant encapsulating process, some new assemblies, such as nanorolls and hollow spindles, have been obtained. Both the nanorolls and hollow spindles showed impressively high catalytic activity for the Suzuki–Miyaura coupling reaction. The catalysts could be reused several times without the loss of the high catalytic efficiency. In addition, the nanorolls could efficiently catalyze the semihydrogenation of alkynes with a higher *Z*-selectivity. Moreover, we think that the high efficiency of the single Pd atom in the Pd-POM is not limited to these two kinds of reactions. A single-atom catalyst with other noble metals in the POMs should be further studied.

## Supplementary Material

Supplementary information

## References

[cit1] Yang X.-F., Wang A., Qiao B., Li J., Liu J., Zhang T. (2013). Acc. Chem. Res..

[cit2] Guo X., Fang G., Li G., Ma H., Fan H., Yu L., Ma C., Wu X., Deng D., Wei M., Tan D., Si R., Zhang S., Li J., Sun L., Tang Z., Pan X., Bao X. (2014). Science.

[cit3] Qiao B., Wang A., Yang X., Allard L. F., Jiang Z., Cui Y., Liu J., Li J., Zhang T. (2011). Nat. Chem..

[cit4] Zhao Z.-J., Chiu C.-c., Gong J. (2015). Chem. Sci..

[cit5] Lin J., Wang A., Qiao B., Liu X., Yang X., Wang X., Liang J., Li J., Liu J., Zhang T. (2013). J. Am. Chem. Soc..

[cit6] Peterson E. J., DeLaRiva A. T., Lin S., Johnson R. S., Guo H., Miller J. T., Hun Kwak J., Peden C. H. F., Kiefer B., Allard L. F., Ribeiro F. H., Datye A. K. (2014). Nat. Commun..

[cit7] Flytzani-Stephanopoulos M., Gates B. C. (2012). Annu. Rev. Chem. Biomol. Eng..

[cit8] Sun S., Zhang G., Gauquelin N., Chen N., Zhou J., Yang S., Chen W., Meng X., Geng D., Banis M. N., Li R., Ye S., Knights S., Botton G. A., Sham T.-K., Sun X. (2013). Sci. Rep..

[cit9] Heiz U., Sanchez A., Abbet S., Schneider W. D. (1999). J. Am. Chem. Soc..

[cit10] Wei H., Liu X., Wang A., Zhang L., Qiao B., Yang X., Huang Y., Miao S., Liu J., Zhang T. (2014). Nat. Commun..

[cit11] Uzun A., Ortalan V., Hao Y., Browning N. D., Gates B. C. (2009). ACS Nano.

[cit12] Herzing A. A., Kiely C. J., Carley A. F., Landon P., Hutchings G. J. (2008). Science.

[cit13] Izarova N. V., Pope M. T., Kortz U. (2012). Angew. Chem., Int. Ed..

[cit14] Yin P., Wang J., Xiao Z., Wu P., Wei Y., Liu T. (2012). Chem.–Eur. J..

[cit15] Huang D., Wang Y. J., Yang L. M., Luo G. S. (2006). Ind. Eng. Chem. Res..

[cit16] Li H., Sun H., Qi W., Xu M., Wu L. (2007). Angew. Chem., Int. Ed..

[cit17] Yin P., Li D., Liu T. (2011). Isr. J. Chem..

[cit18] Nisar A., Lu Y., Zhuang J., Wang X. (2011). Angew. Chem., Int. Ed..

[cit19] Nisar A., Zhuang J., Wang X. (2011). Adv. Mater..

[cit20] Kim S.-W., Kim M., Lee W. Y., Hyeon T. (2002). J. Am. Chem. Soc..

[cit21] Teschner D., Borsodi J., Wootsch A., Révay Z., Hävecker M., Knop-Gericke A., Jackson S. D., Schlögl R. (2008). Science.

[cit22] Mori K., Hara T., Mizugaki T., Ebitani K., Kaneda K. (2004). J. Am. Chem. Soc..

[cit23] Izarova N. V., Banerjee A., Kortz U. (2011). Inorg. Chem..

[cit24] He P., Xu B., Liu H., He S., Saleem F., Wang X. (2013). Sci. Rep..

[cit25] He P., Xu B., Wang P.-p., Liu H., Wang X. (2014). Adv. Mater..

[cit26] Grunwaldt J.-D., Vegten N. v., Baiker A. (2007). Chem. Commun..

[cit27] Choi M., Lee D.-H., Na K., Yu B.-W., Ryoo R. (2009). Angew. Chem., Int. Ed..

[cit28] Xu B., Yang H., Zhou G., Wang X. (2014). Sci. China Mater..

[cit29] Maegawa T., Kitamura Y., Sako S., Udzu T., Sakurai A., Tanaka A., Kobayashi Y., Endo K., Bora U., Kurita T., Kozaki A., Monguchi Y., Sajiki H. (2007). Chem.–Eur. J..

[cit30] Plault L., Hauseler A., Nlate S., Astruc D., Ruiz J., Gatard S., Neumann R. (2004). Angew. Chem., Int. Ed..

[cit31] Qi W., Wang Y., Li W., Wu L. (2010). Chem.–Eur. J..

[cit32] Wu J., Wang K. J., Li Y., Yu P. (2011). Adv. Mater. Res..

[cit33] Brunet J. J., Caubere P. (1984). J. Org. Chem..

[cit34] Yan M., Jin T., Ishikawa Y., Minato T., Fujita T., Chen L.-Y., Bao M., Asao N., Chen M.-W., Yamamoto Y. (2012). J. Am. Chem. Soc..

