# Supplementary material for: Surfactant encapsulated palladium-polyoxometalates: controlled assembly and their application as single-atom catalysts
Source: Chem Sci. 2015 Oct 26;7(2):1011–5. doi: 10.1039/c5sc03554f (PMC5954849; doi:10.1039/c5sc03554f)

Electronic Supplementary Material (ESI) for Chemical Science.
This journal is © The Royal Society of Chemistry 2015

Surfactants Encapsulated Palladium-Polyoxometalates: Controlled Assembly and Their Application as Single-atom Catalysts

Peilei He,[a] Biao Xu,[a] Xiaobin Xu,[a] Li Song,[b] and Xun Wang[a]*

a Department of Chemistry, Tsinghua University, Beijing, 100084, China

E-mail: wangxun@mail.tsinghua.edu.cn

b National Synchrotron Radiation Laboratory, University of Science and Technology of China, Hefei, 230029, China

Experimental Section

The procedure leading to Surfactant-Encapsulated POMs: [$(CTA)_x(TBA)_{(16-x)}Pd_2(\alpha_2\text{-}P_2W_{17}O_{61})_2$ for example]: CTA. Br (0.15 mmol) and TBA. Br (0.15 mmol) were added into $CHCl_3$ (15 mL). Then this organic phase was dropwise added into the aqueous solution of POMs ($K_{15}[Pd_2(\alpha_2\text{-}P_2W_{17}O_{61}H_{0.5})_2]$, 0.019 mmol, 20 mL) with stirring. After 1 hour, the organic phase was placed in a petri dish. Finally, the SEP product (a yellow thin-film) was collected by evaporation of the $CHCl_3$. $(DTA)_x(TEA)_{(16-x)}Pd_2(\alpha_2\text{-}P_2W_{17}O_{61})_2$, $(CTA)_3(TBA)_3P_2W_{18}O_{62}$ and $(CTA)_x(TBA)_{(10-x)}P_2W_{17}O_{61}$ were got by the same method. However, the only difference is that $(DTA)_8(TEA)_8Pd_2(\alpha_2\text{-}P_2W_{17}O_{61})_2$ was formed in the aqueous solution. The ratio between the two kinds of surfactants is close to 1 to 1 in the final products.

The preparation of catalyst: The SEPs (0.1 mg) of $(CTA)_x(TBA)_{(16-x)}Pd_2(\alpha_2\text{-}P_2W_{17}O_{61})_2$ was dissolved in chloroform (20 mL). Then acetone (10 mL) was added into the chloroform solution. Then the mixed solution was put in the fume hood. After the organic solvents were completely evaporated, the catalyst of nanoroll sample was got. On the other hand, the hollow spindle was formed during the surfactant-encapsulating process. The hollow spindle is in the aqueous phase of the two-phase system. And the catalyst of hollow spindle sample was got after the water was completely evaporated.

General procedure for Suzuki-Miyaura reaction: Aryl halide (0.5 mmol), phenylboronic acid (0.75 mmol) and $[(CTA)_8(TBA)_8Pd_2(\alpha_2\text{-}P_2W_{17}O_{61})_2$ were added into 1 mL ethanol. K_2CO_3 (1.5 mmol) and TBAB (0.12 mmol) were dissolved in 1 mL deionized water. Then the aqueous solution was mixed with the ethanol solution under continuous magnetic stirring. This reaction system temperature was stabilized at 30 ^{o}C for 20 mins. Finally, the solution was extracted with ethyl acetate and concentrated by rotary evaporator. In the recycle experiments, the catalyst was recovered by centrifugation. The isolated yield was got by column chromatography on silica gel.

General procedure for the recovery of catalysts: First, ethyl acetate was added into the reaction system after the reaction. Then the mixed solution was taken to centrifugation. The precipitate (the recyclable catalyst) was washed by water and ethanol to remove the substrates.

General Procedure for Semihydrogenation reaction: To a $CHCl_3$ solution (8mg, 2 mL) of $[(CTA)_8(TBA)_8Pd_2(\alpha_2\text{-}P_2W_{17}O_{61})_2$ were added deionized water (10 μL), dimethylphenylsilane (0.2 mmol) and alkynes (0.1 mmol) subsequently at room temperature. The mixture was stirred at 25 ^{o}C for 30 mins. In the recycle experiments, the catalyst was recovered by centrifugation. Then the solution was extracted with ethyl acetate and concentrated by rotary evaporator. ^{1}H NMR yield was obtained using CH_2Br_2 as an internal standard.

Characterization: Transmission electron microscope (TEM) was characterized on a Hitachi H-7700 at 100KV. XPS were performed on were recorded on a PHI Quantera SXM spectrometer and all the peak values were calibrated according to C_{1s} peak (284.6 eV). And a Tecnai G2 F20 S-Twin high-resolution transmission electron microscope at 200KV (HRTEM) was used to characterize the sample. IR was carried out on a Perkin Elmer Spectrum. Small-angle X-ray diffraction (SAXRD) characterization was carried on a Bruker D8 X-ray diffractometer using Cu Kα radiation (λ= 0.15418 nm). And the ^{31}P NMR spectrum was got from JEOL ECA-600 NMR spectrometer (600 MHz, D_2O). The ^{1}H NMR and ^{13}C NMR spectrums were got from JEOL ECA-400 NMR spectrometer (400 MHz, $CDCl_3$). X-ray absorption spectroscopy experiments were performed at Shanghai Synchrotron Radiation Facility (SSRF), Shanghai Institute of Applied physics (SINAP), China.

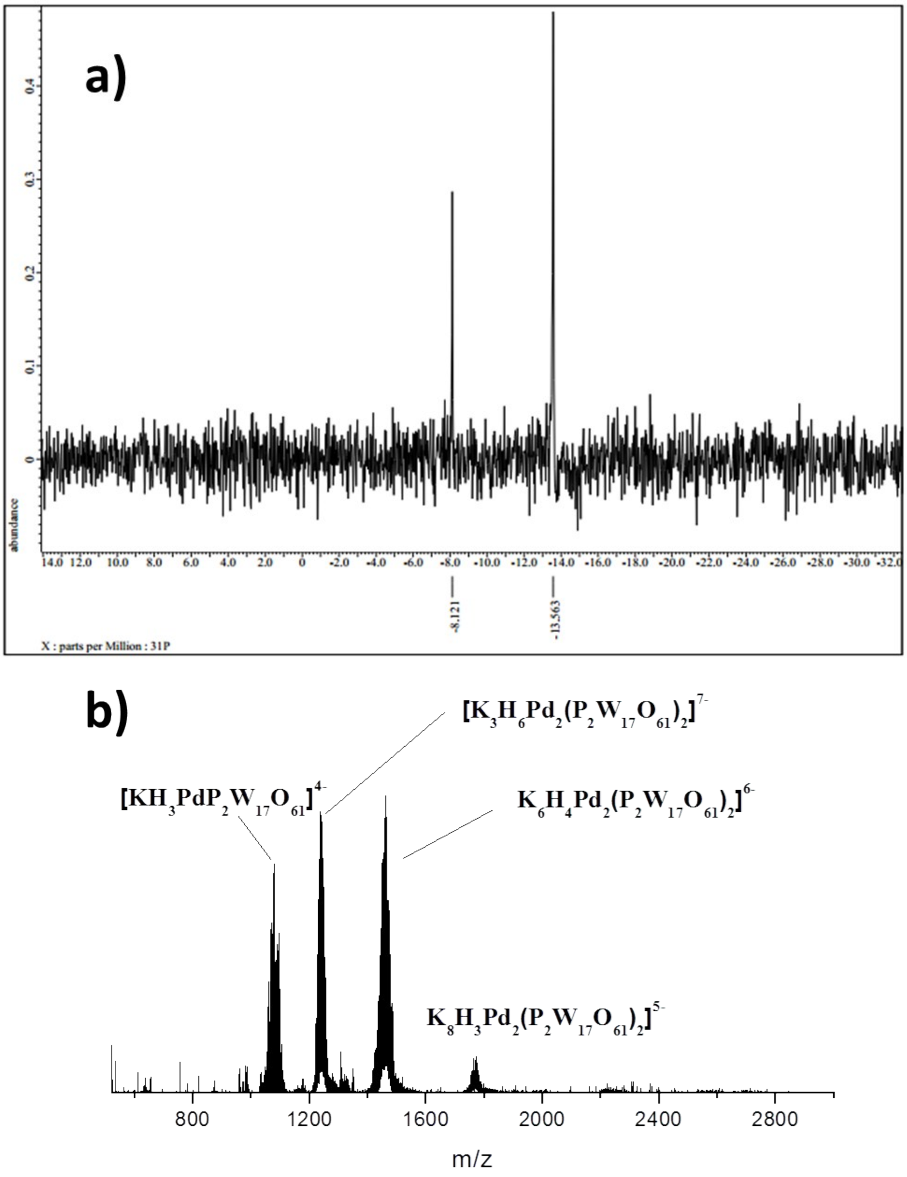

Figure S1. a) ^{31}P NMR and b) ESI mass spectrum of the Pd-Dawson structure.

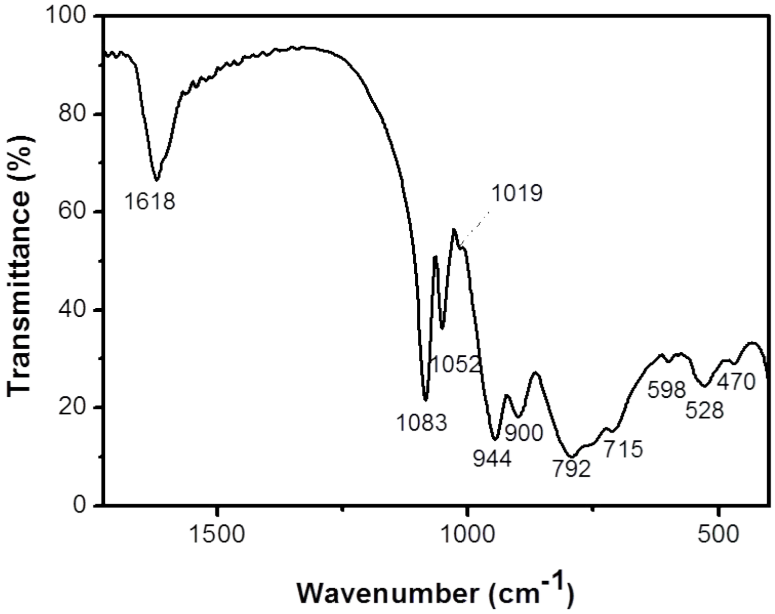

Figure S2. The IR spectrum of the Pd-Dawson structure. IR (2% KBr pellets, ν/cm^{-1}): 1618 (s), 1083 (m), 1052 (m), 1019 (m), 944 (s), 900 (m), 792 (sh), 715 (s), 598 (w), 528 (m), 470(w).

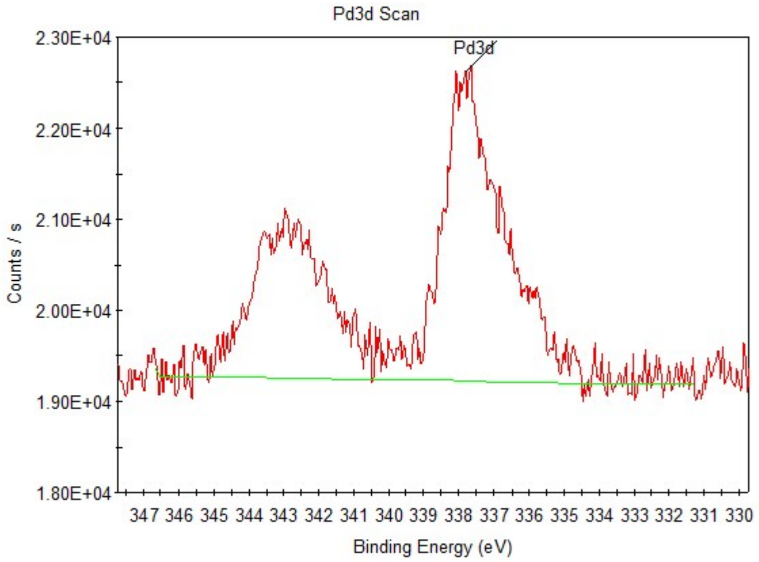

Figure S3. XPS spectrum of the Pd-Dawson structure.

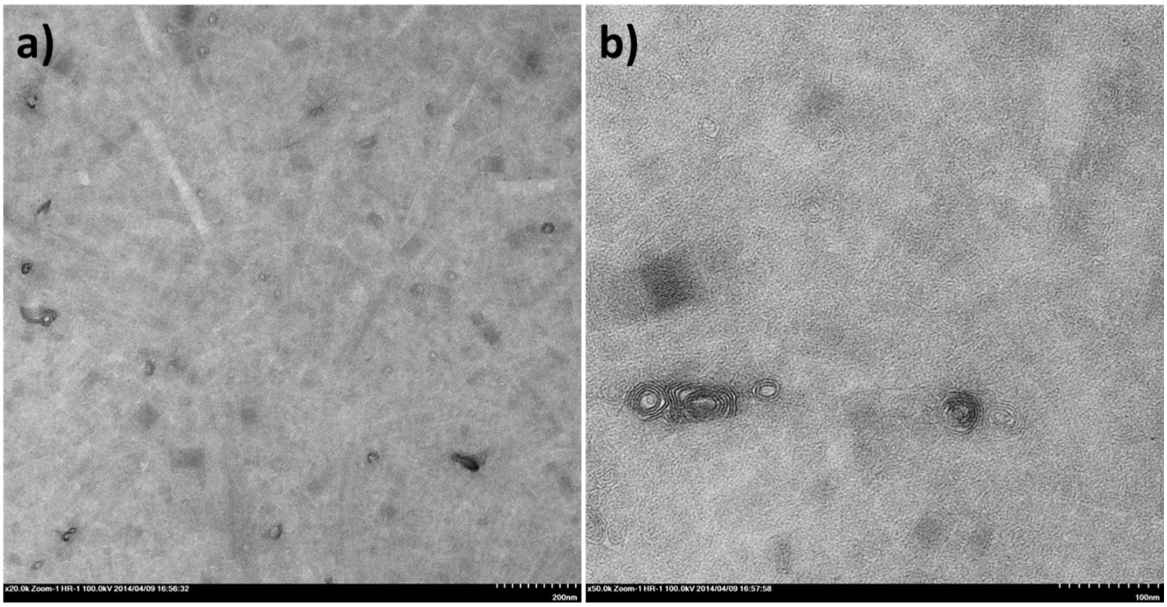

Figure S4. a) TEM images when the film was dispersed in chloroform. b) The magnified image of a).

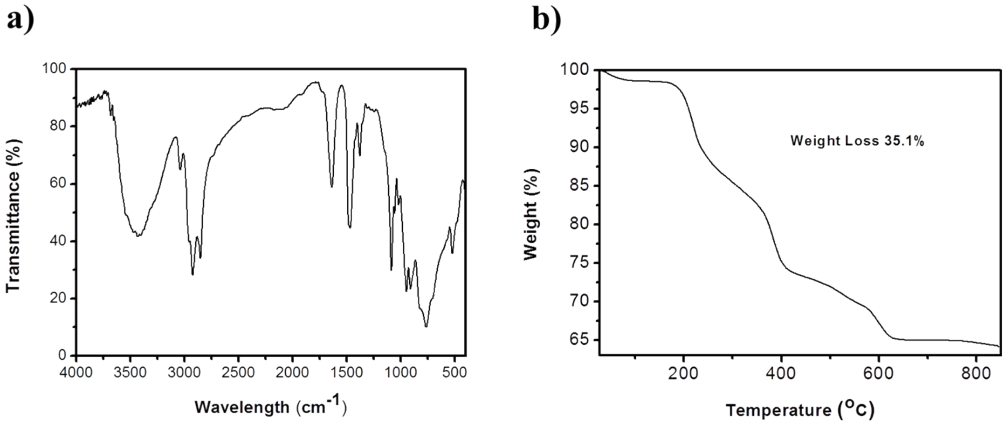

Figure S5. a) FTIR spectrum and b) TGA result of the nanorolls.

sample	N content (%)	C content (%)	H content (%)
nanoroll	1.984	24.710	4.730
hollow spindle	1.548	12.600	2.839

Figure S6. C, H, N elemental analysis of the nanocoils and hollow spindles

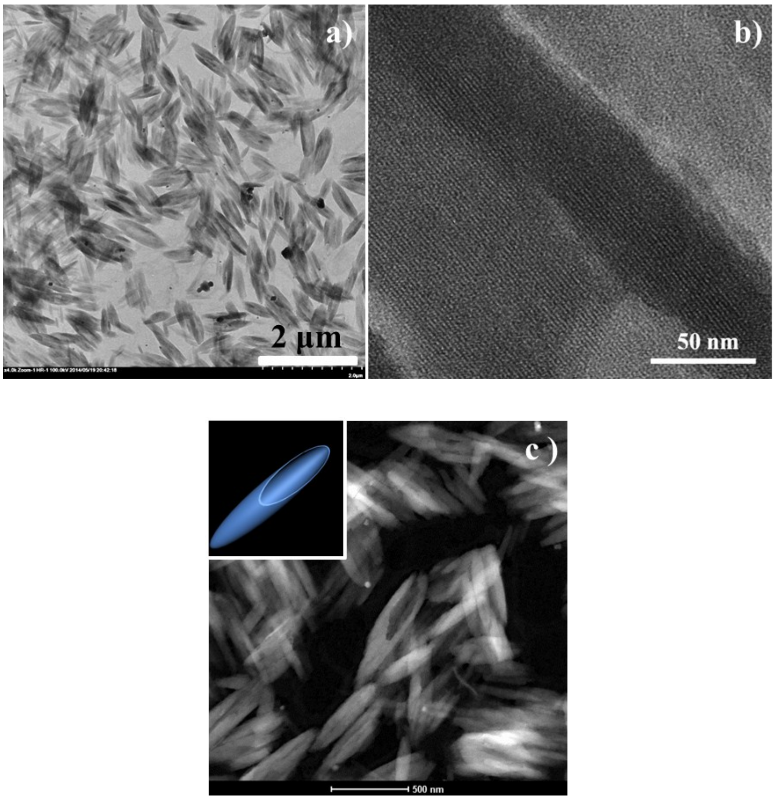

Figure S7. a) TEM image of the hollow spindles. b) Magnified TEM image of the hollow spindle. c) STEM image and a scheme of the hollow spindle.

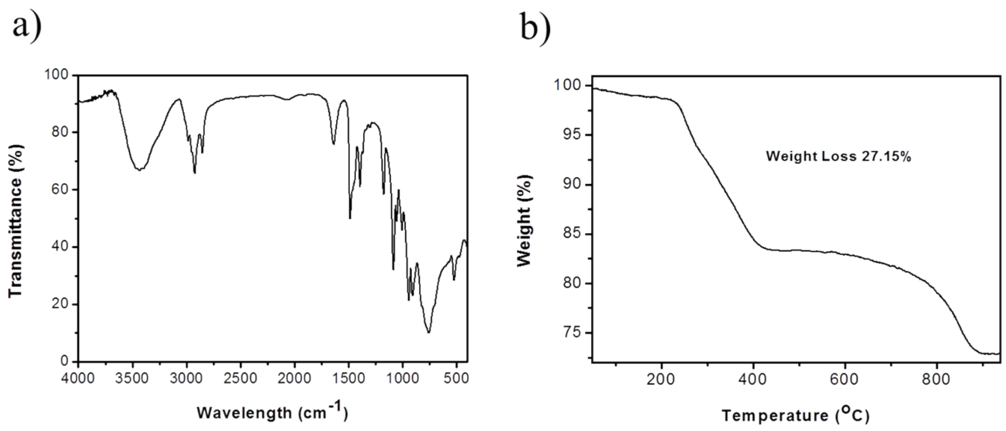

Figure S8. a) FTIR spectrum and b) TGA result of the hollow spindles.

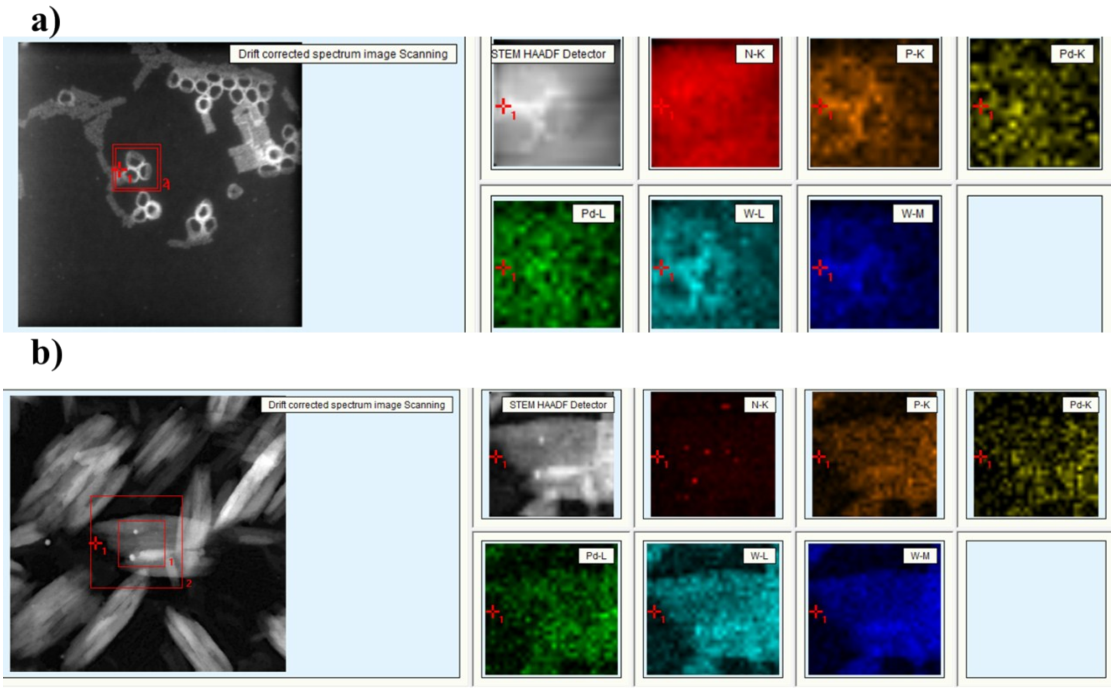

Figure S9. a) EDX mapping of the nanocoils. b) EDX mapping of the hollow spindles.

sample	Pd content (%)
nanocoils	1.704
hollow spindles	2.328

Figure S10. ICP results of the nanocoils and hollow spindles.

Table S1: The scope of the Suzuki-Miyaura Coupling reaction.[a]

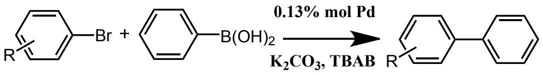

entry	R	catalyst	Yield [%][b]	TON	TOF [h^{-1}]
1		nanoroll	96	750	2250
2	4-$COCH_3$	spindle	92	719	2156
3		Pd-POM	36	281	844
4		Pd/C	8	62	188
5	4-OCH_3	nanoroll	81	633	1898
6		spindle	88	688	2063
7	4-CH_3	nanoroll	86	672	2016
8		spindle	79	617	1852
9	4-$COOC_2H_5$	nanoroll	97	758	2273
10		spindle	95	742	2226
11	4-NO_2	nanoroll	92	719	2156
12		spindle	92	719	2156
13	3-CHO	nanoroll	85	664	1992
14		spindle	90	703	2109

[a] The reactions were performed with phenylbromide (0.5 mmol), phenylboronic acid (0.75 mmol), K_2CO_3 (1.5 mmol), TBAB (0.12 mmol), ethanol (1 mL) and water (1 mL) in the presence of different catalysts at 30 °C for 20 min. [b] Isolated yield.

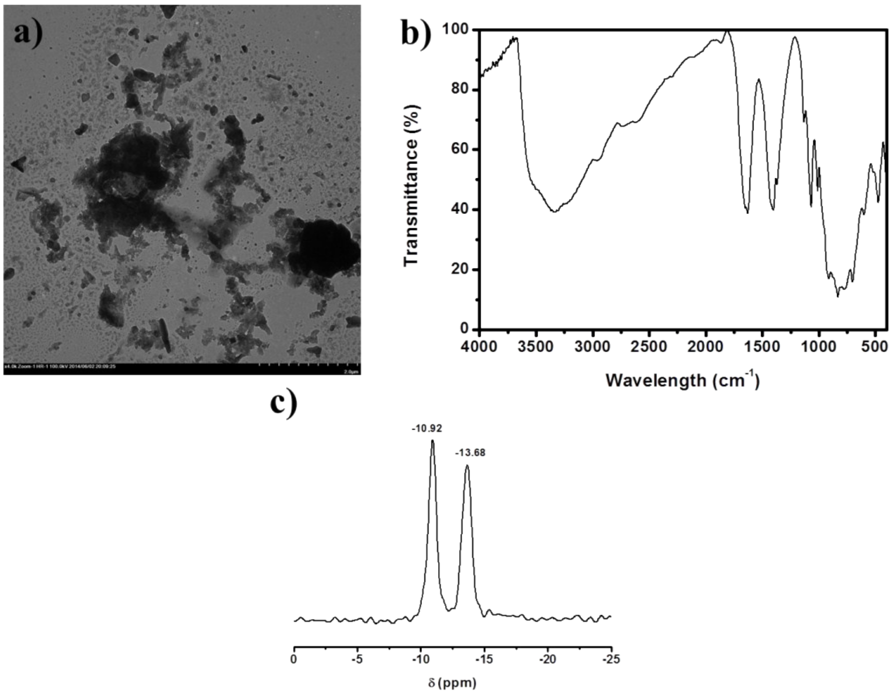

Figure S11. a) TEM image and b) FTIR spectrum and c) MAS ^{31}P NMR spectrum of nanorolls after

Suzuki-Miyaura Coupling reaction.

	recovery percentage of catalyst	
cycle 1	96%	
cycle 2	without CTAB	with CTAB
	90%	96%

Figure S12. The recovery percentage of catalyst after Suzuki-Miyaura Coupling reaction.

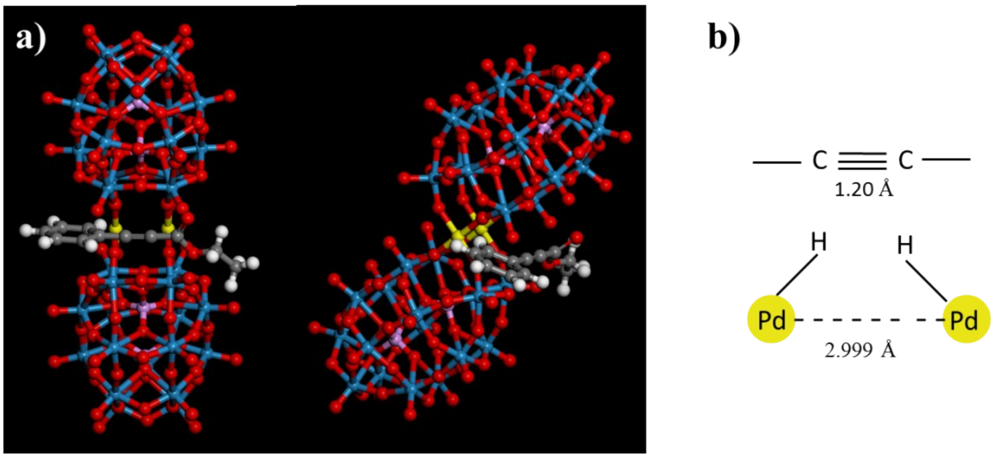

Figure S13. a) The scheme of front view and side view when the alkyne is in the center of Pd-POM. b) A simple sketch of the catalytic center.

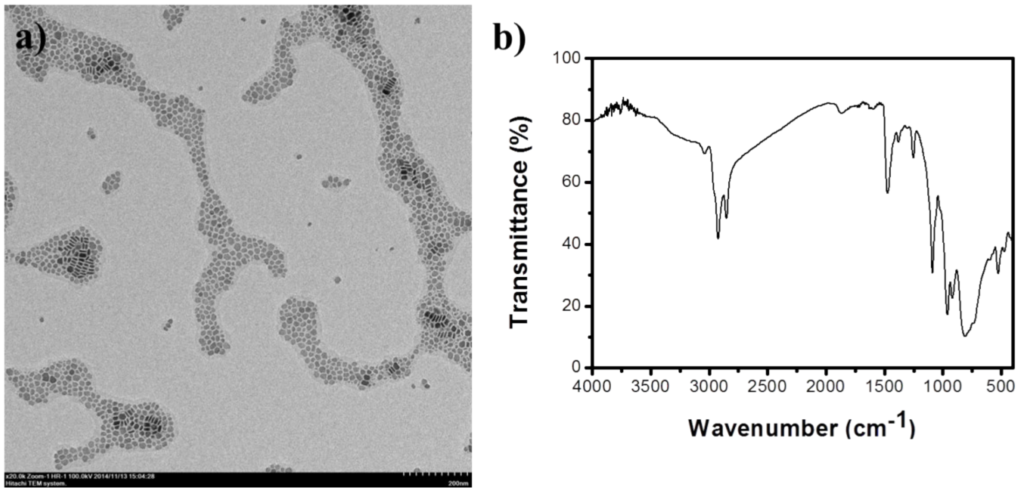

Figure S14. a) TEM image and b) FTIR spectrum of nanocoils after Semihydrogenation reaction.

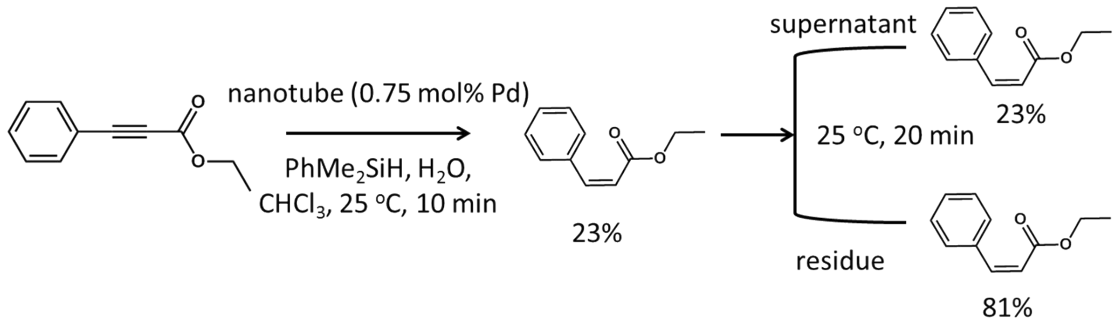

Figure S15. The leaching experiments.

^{1}H and ^{13}C NMR spectra of the product of Suzuki-Miyaura Coupling reaction

4-acetylbiphenyl (entry 1, 2 and 3 in Table S1)

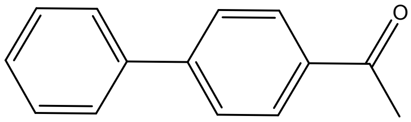

^{1}H-NMR spectra:

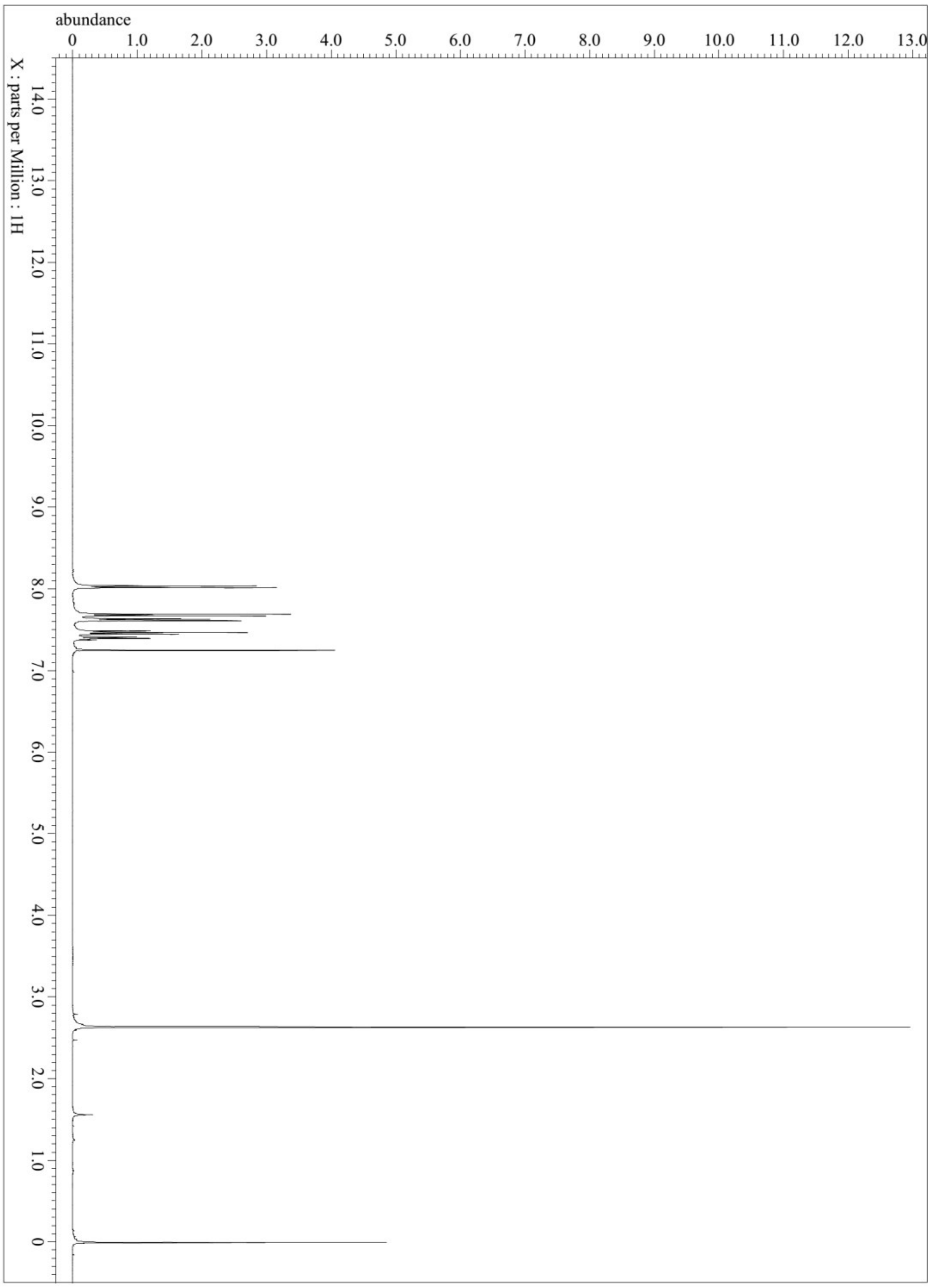

^{13}C NMR spectra

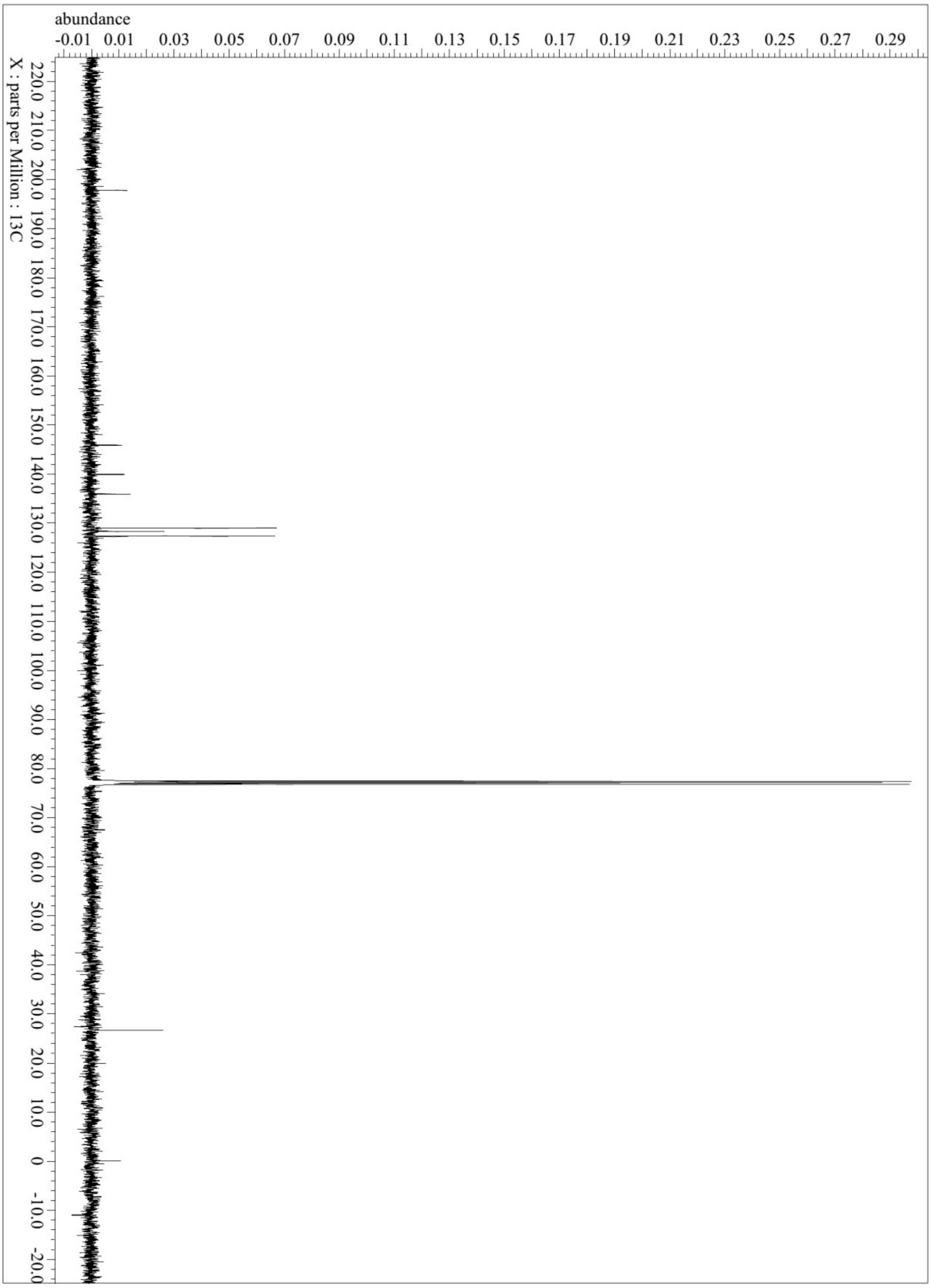

4-methoxylbiphenyl (entry 4 and 5 in Table S1)

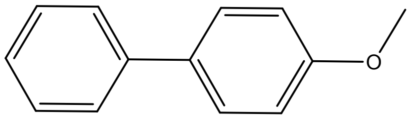

^{1}H-NMR spectra:

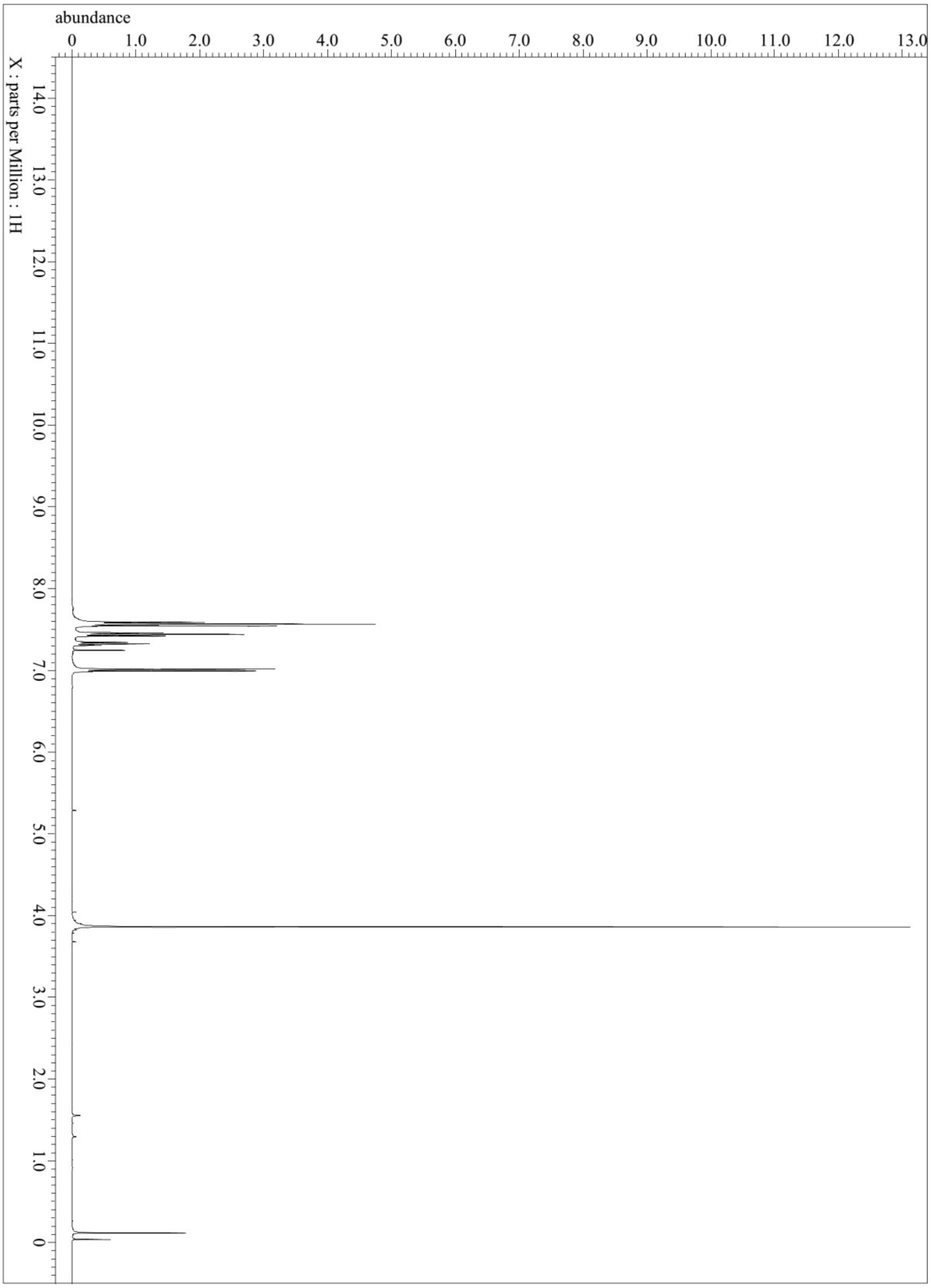

^{13}C NMR spectra:

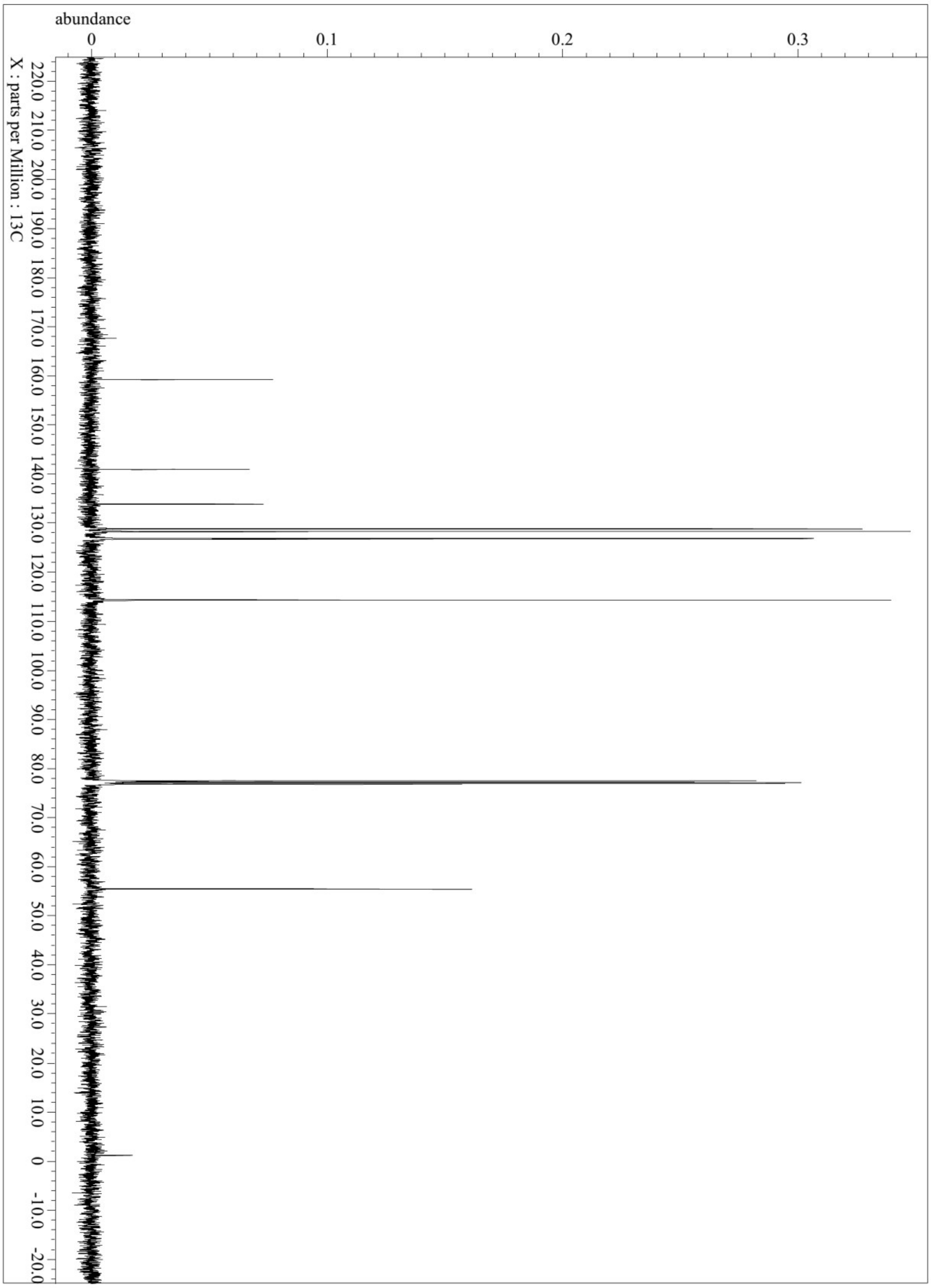

4-methylbiphenyl (entry 6 and 7 in Table S1)

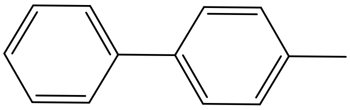

^{1}H-NMR spectra:

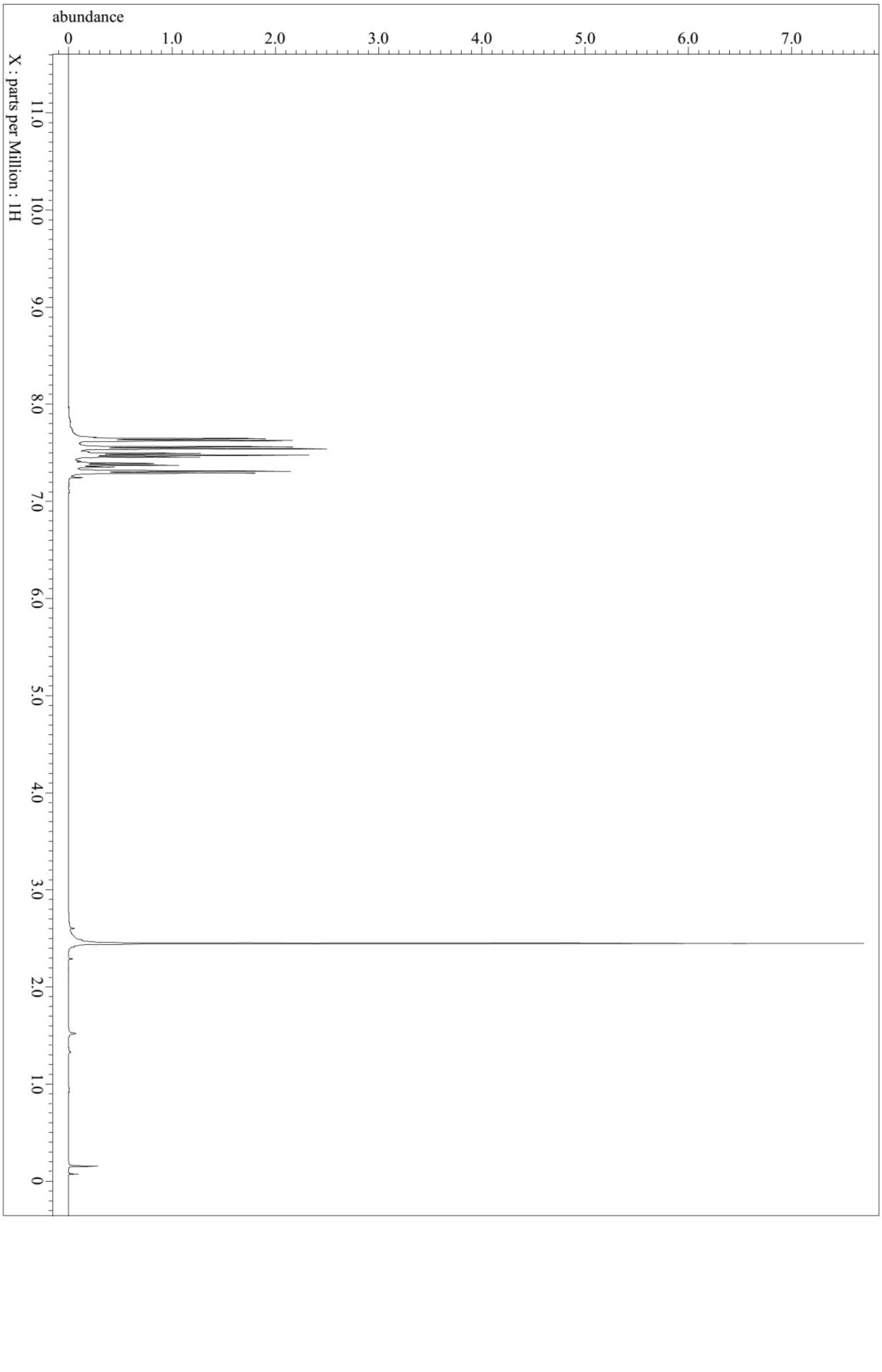

^{13}C NMR spectra:

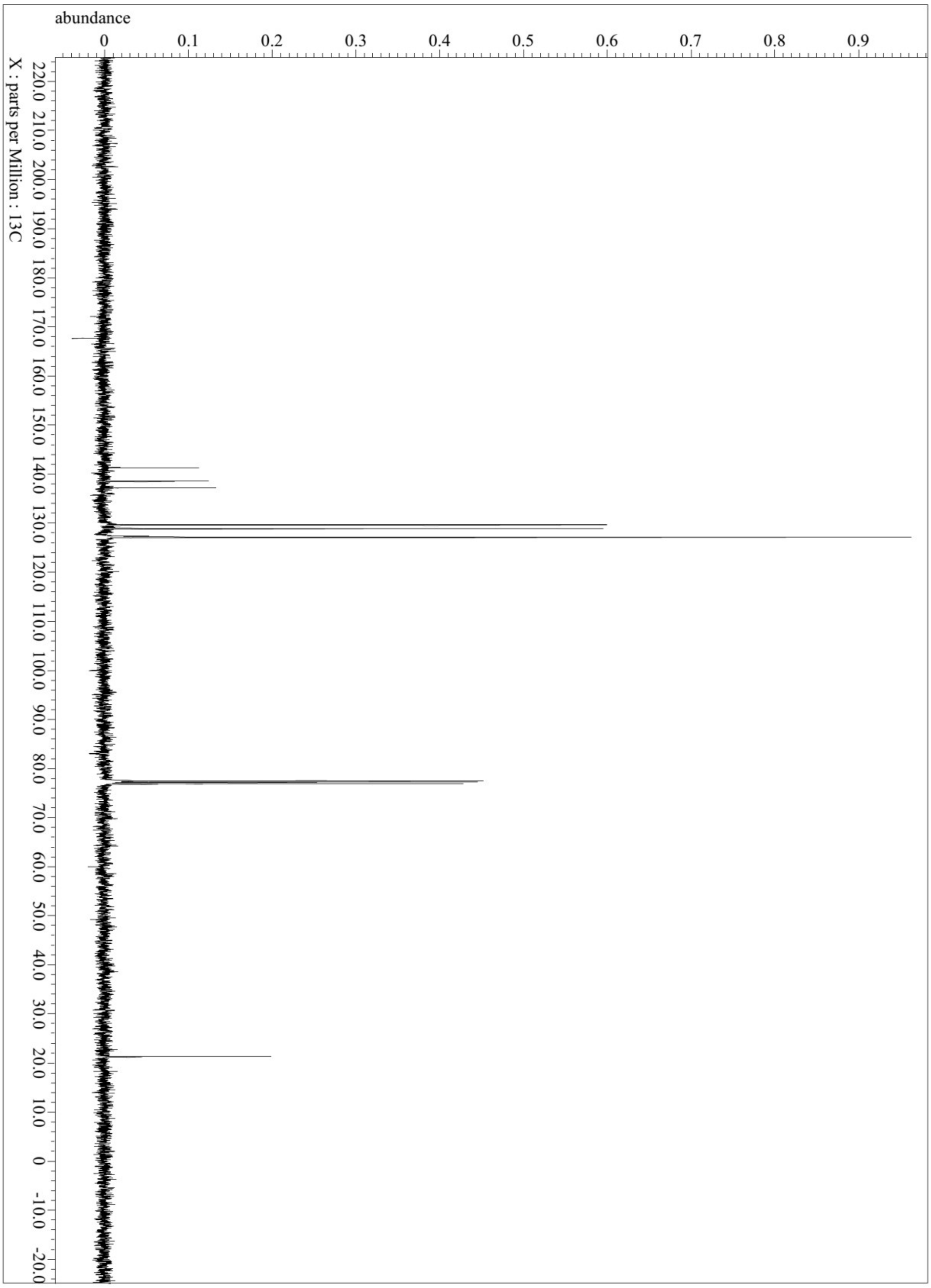

4-ethoxylcarbonylbiphenyl (entry 8 and 9 in Table S1)

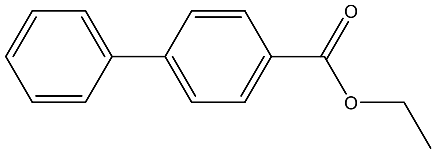

^{1}H-NMR spectra:

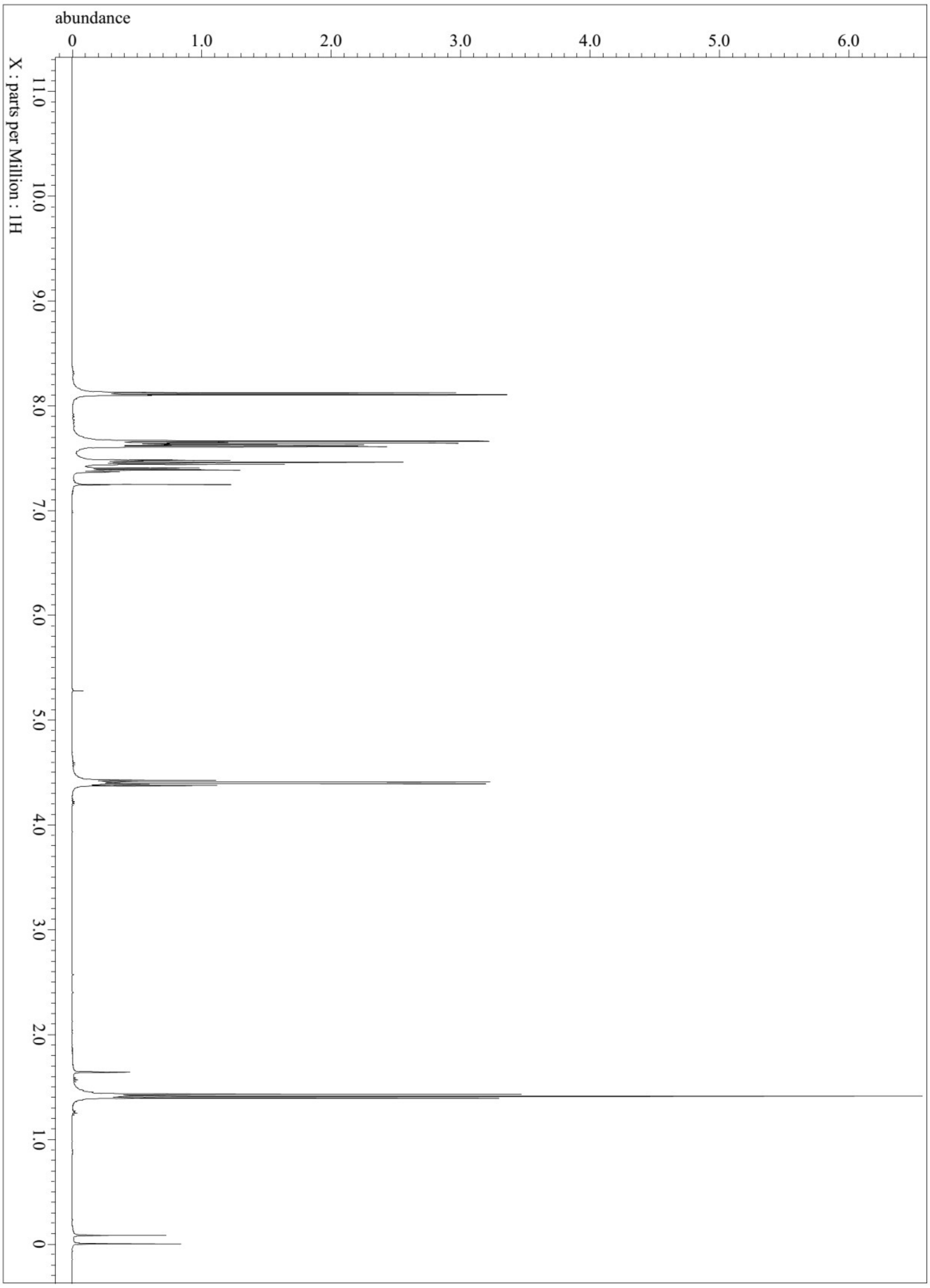

^{13}C NMR spectra:

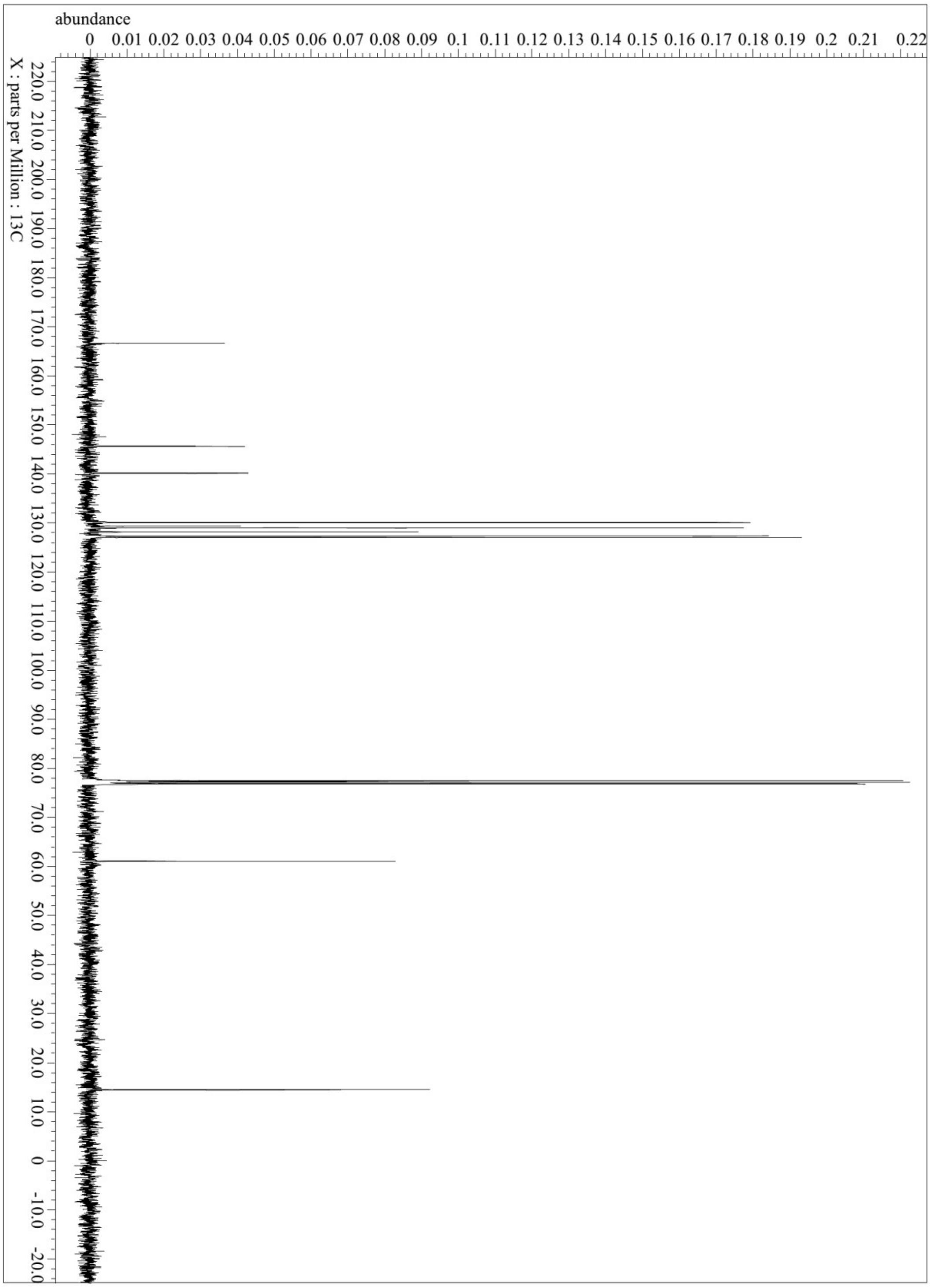

4-nitrobiphenyl (entry 10 and 11 in Table S1)

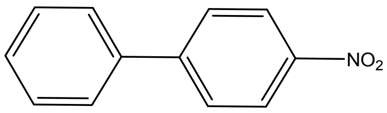

^{1}H-NMR spectra:

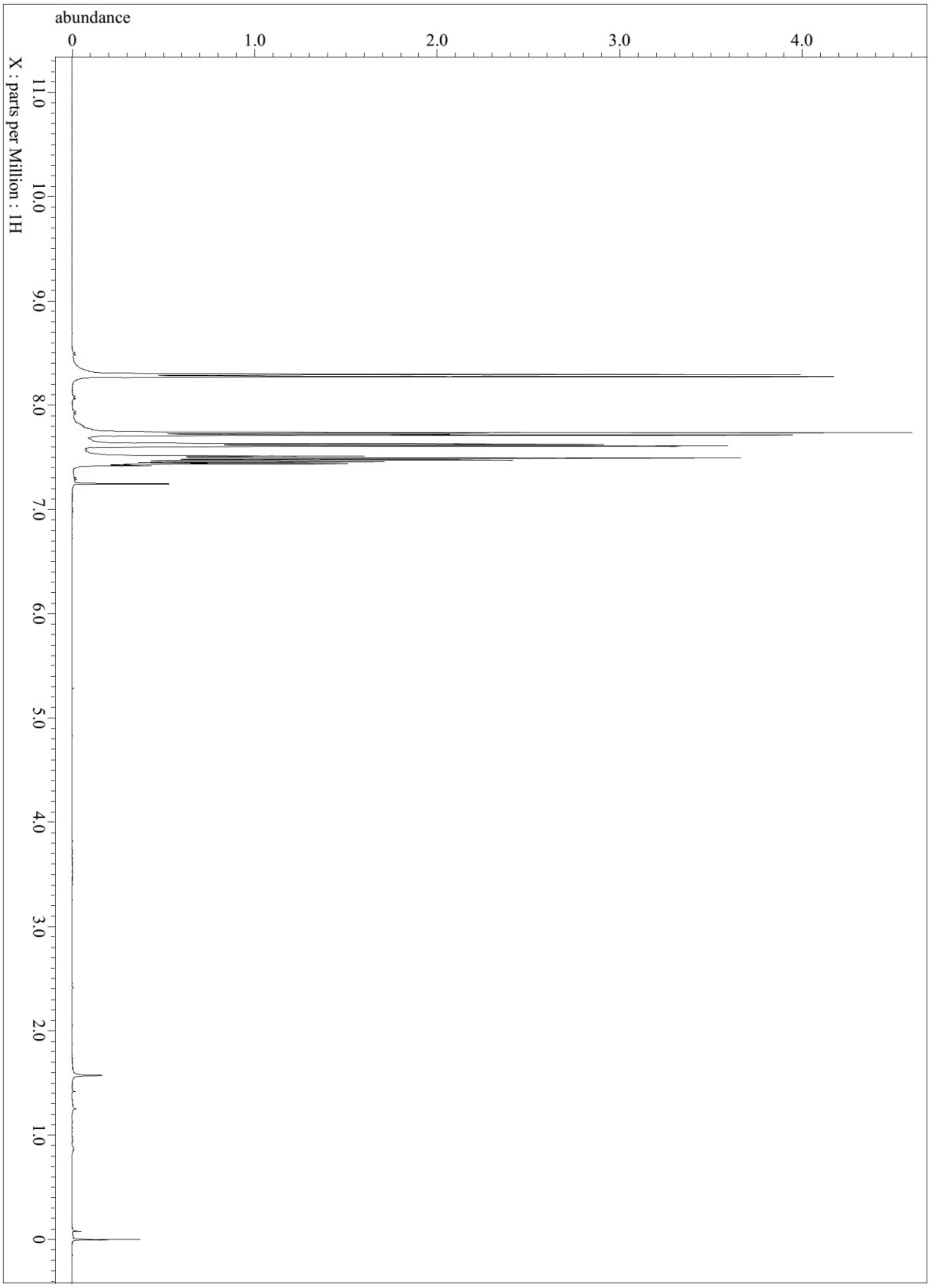

^{13}C NMR spectra:

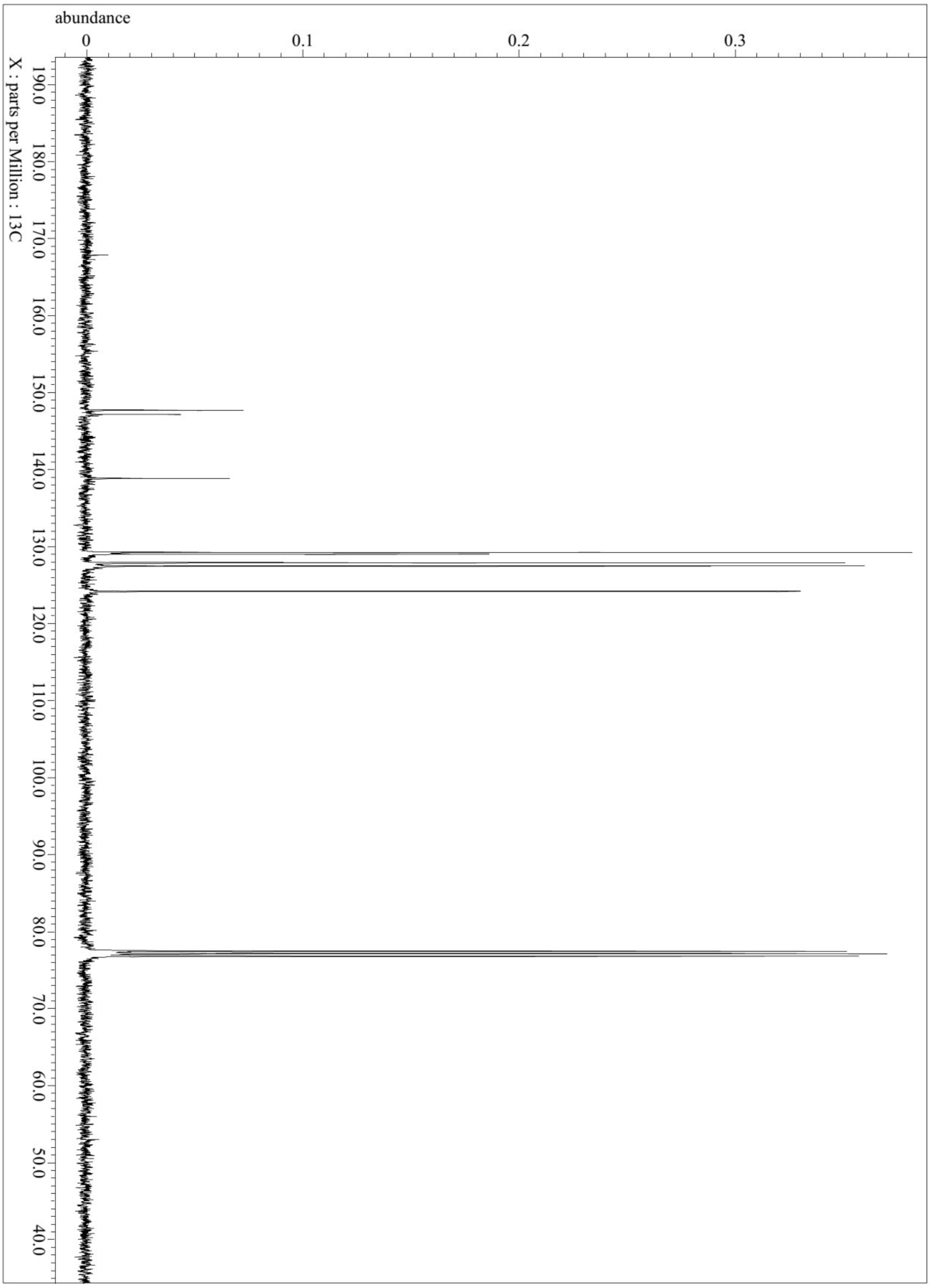

3-biphenylcarboxaldehyde (entry 12 and 13 in Table S1)

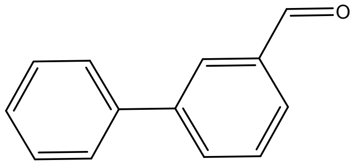

^{1}H-NMR spectra:

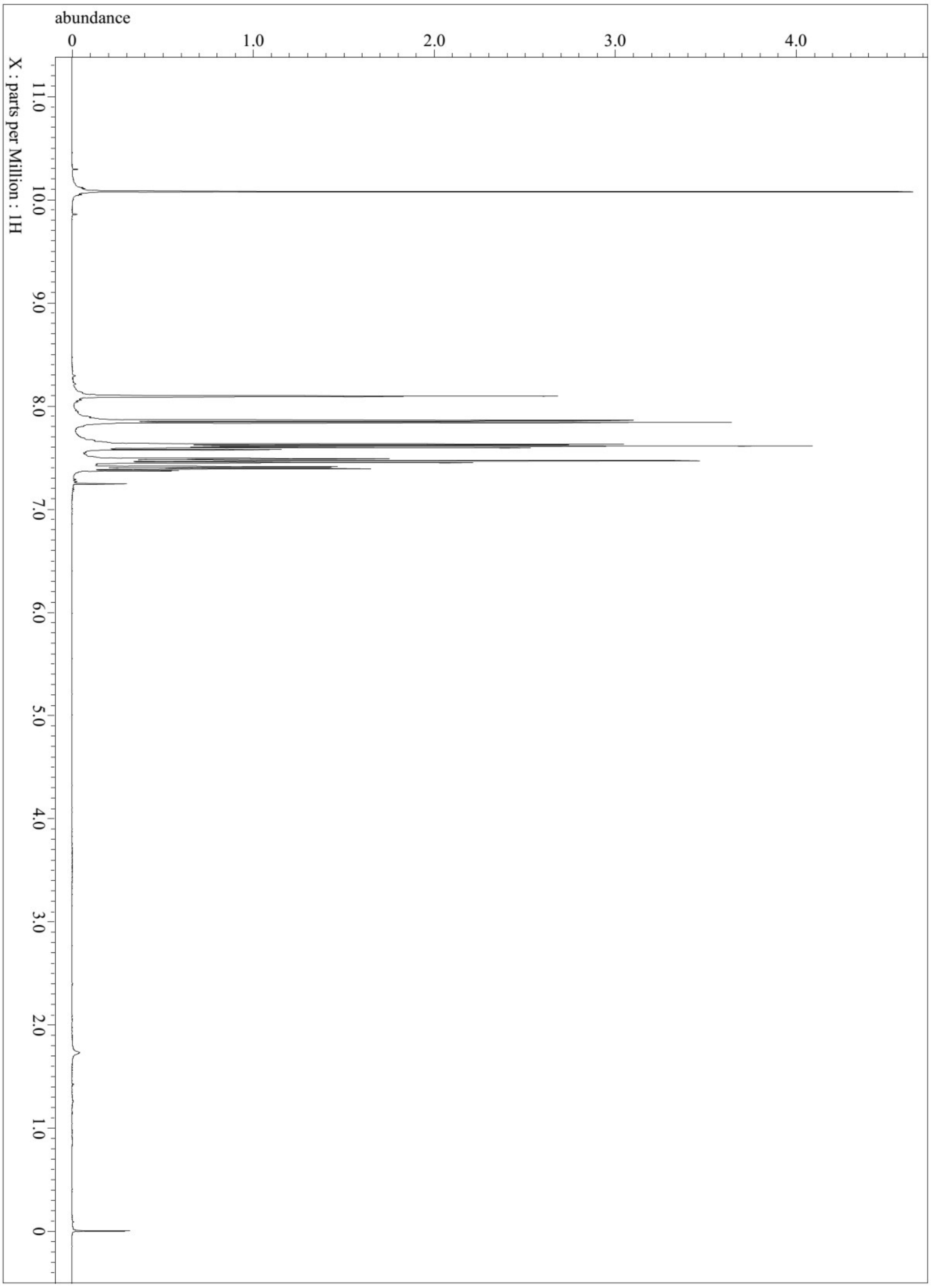

^{13}C NMR spectra:

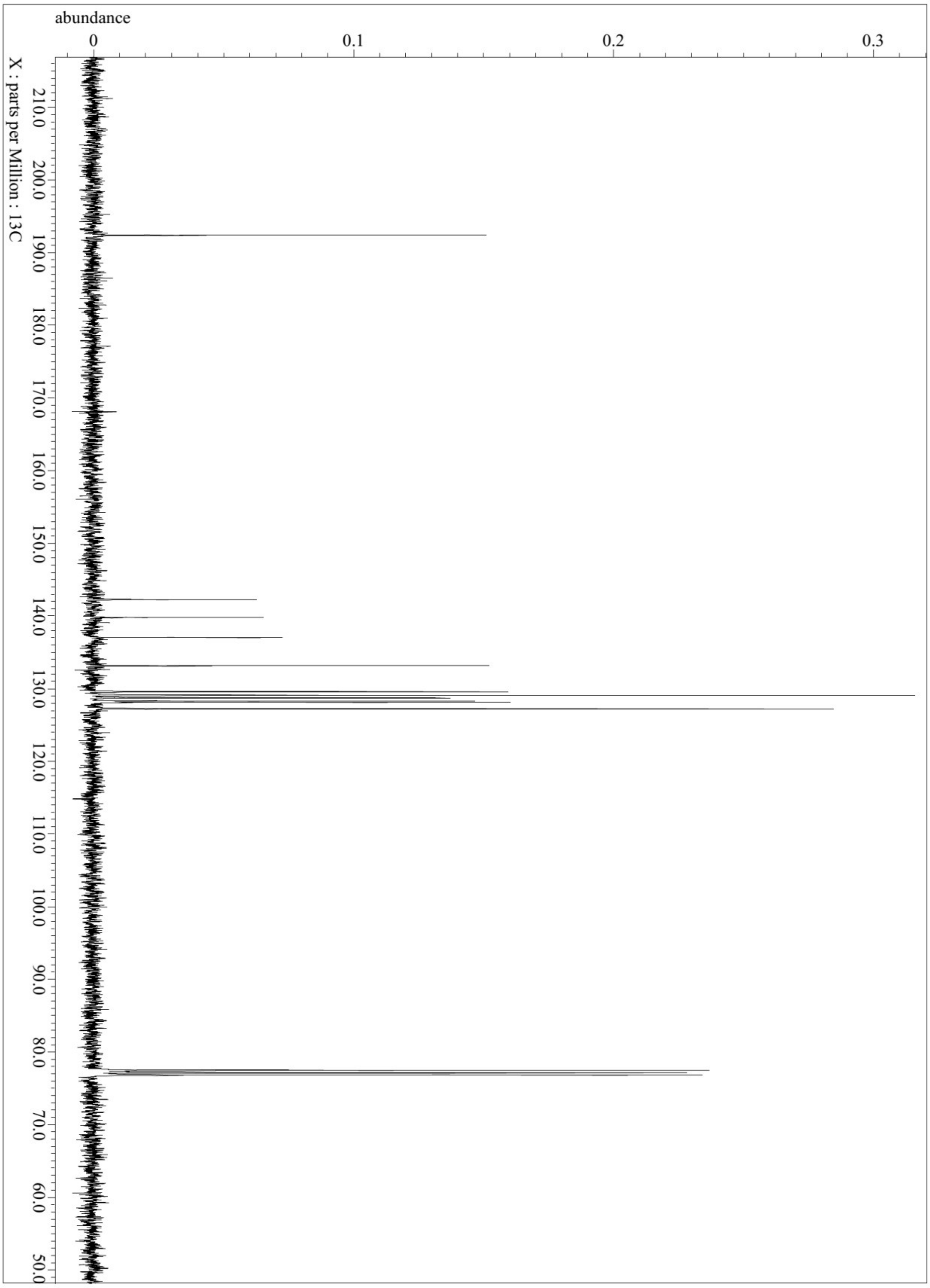

Supplement: Supplementary file 1 [file SC-007-C5SC03554F-s001.pdf]
